# Liquid-Phase Hydrogenation of 1-Phenyl-1-propyne on the Pd_1_Ag_3_/Al_2_O_3_ Single-Atom Alloy Catalyst: Kinetic Modeling and the Reaction Mechanism

**DOI:** 10.3390/nano11123286

**Published:** 2021-12-03

**Authors:** Alexander V. Rassolov, Igor S. Mashkovsky, Galina N. Baeva, Galina O. Bragina, Nadezhda S. Smirnova, Pavel V. Markov, Andrey V. Bukhtiyarov, Johan Wärnå, Alexander Yu. Stakheev, Dmitry Yu. Murzin

**Affiliations:** 1 N.D. Zelinsky Institute of Organic Chemistry RAS, Leninsky Prospect 47, 119991 Moscow, Russia; brandon_hitt@mail.ru (A.V.R.); bg305@ioc.ac.ru (G.N.B.); bragina@ioc.ac.ru (G.O.B.); felisine@gmail.com (N.S.S.); markovp@mail.ru (P.V.M.); 2 Synchrotron Radiation Facility SKIF, Boreskov Institute of Catalysis SB RAS, Nikol’skiy Prospekt 1, 630559 Kol’tsovo, Russia; avb@catalysis.ru; 3 Laboratory of Industrial Chemistry and Reaction Engineering, Faculty of Science and Engineering, Åbo Akademi University, FI-20500 Turku, Finland; johan.warna@abo.fi

**Keywords:** single-atom alloy catalyst, alkyne, alkene selectivity, hydrogenation, 1-phenyl-1-propyne, kinetic modeling

## Abstract

This research was focused on studying the performance of the Pd_1_Ag_3_/Al_2_O_3_ single-atom alloy (SAA) in the liquid-phase hydrogenation of di-substituted alkyne (1-phenyl-1-propyne), and development of a kinetic model adequately describing the reaction kinetic being also consistent with the reaction mechanism suggested for alkyne hydrogenation on SAA catalysts. Formation of the SAA structure on the surface of PdAg_3_ nanoparticles was confirmed by DRIFTS-CO, revealing the presence of single-atom Pd_1_ sites surrounded by Ag atoms (characteristic symmetrical band at 2046 cm^−1^) and almost complete absence of multiatomic Pd_n_ surface sites (<0.2%). The catalyst demonstrated excellent selectivity in alkyne formation (95–97%), which is essentially independent of P(H_2_) and alkyne concentration. It is remarkable that selectivity remains almost constant upon variation of 1-phenyl-1-propyne (1-Ph-1-Pr) conversion from 5 to 95–98%, which indicates that a direct alkyne to alkane hydrogenation is negligible over Pd_1_Ag_3_ catalyst. The kinetics of 1-phenyl-1-propyne hydrogenation on Pd_1_Ag_3_/Al_2_O_3_ was adequately described by the Langmuir-Hinshelwood type of model developed on the basis of the reaction mechanism, which suggests competitive H_2_ and alkyne/alkene adsorption on single atom Pd_1_ centers surrounded by inactive Ag atoms. The model is capable to describe kinetic characteristics of 1-phenyl-1-propyne hydrogenation on SAA Pd_1_Ag_3_/Al_2_O_3_ catalyst with the excellent explanation degree (98.9%).

## 1. Introduction

Selective hydrogenation of alkynes to corresponding alkenes is of immense importance being widely used in the production of monomers, fine chemicals, and pharmaceuticals [1,2]. Hydrogenation of acetylenic compounds is widely applied in the purification of ethylene produced by steam cracking of naphtha, because the presence of the traces of acetylene in ethylene leads to irreversible poisoning of metallocene polymerization catalysts due to its strong adsorption on the catalyst active sites. Moreover, acetylene admixture can deteriorate the properties of the final polymers [3,4,5]. Another example is the selective hydrogenation of alkynols used to synthesize precursors for the production of vitamin E and linalool [6,7,8].

Various catalysts have been explored for the selective hydrogenation of alkynes, with palladium catalysts being a primary choice due to its favorable activity, even if selectivity to alkene is not sufficient enough. Thus, the undesired overhydrogenation and oligomerization may be induced by the presence of bulk PdH_x_ in the subsurface region of Pd nanoparticles, decreasing the desired selectivity and leading to catalyst deactivation [9]. Bimetallic Pd-M catalysts demonstrate significantly better performance; however, their selectivity is also limited by a nonuniform structure of active sites on the surface of metal nanoparticles. Active sites nonuniformity stems from several factors: (1) the active site can include a different number of surface atoms; (2) the atoms located on the edges, faces, or planes of a metal nanoparticle have different degrees of coordination unsaturation, etc. As a result, adsorption and catalytic characteristics of active sites differ significantly, affecting selectivity [10]. Size effects and structure sensitivity was previously reported for hydrogenation of phenylacetylene and diphenylacetylene [11,12,13].

Currently, the most promising solution to this problem is the concept of single-atom alloy catalysts, which are at the forefront of modern catalysis [14]. As a rule, these are bimetallic systems with a highly-ordered surface structure of active sites being formed through isolation of individual atoms of the active component (Pt, Rh, Pd) by atoms of an inactive or a less active metal (Au, Ag, Cu) [15,16,17]. The thermodynamic stability of the SAA structure makes it possible to regenerate SAAs catalyst at high temperatures (>500–700 °C). This characteristic allows them to be used not only in laboratory practice but also in industry.

The favorable selectivity of SAA catalysts in hydrogenation of alkynes or conjugated dienes has been demonstrated repeatedly. In alkyne hydrogenation excellent selectivity of SAAs toward alkene formation was reported for PdZn, PdCu, PdAu and PdAg compositions [14,18,19,20,21,22,23]. The efficiency of PtCu in hydrogenation of 1,3-butadiene was discovered by Sykes et al. [16,24] and theoretically confirmed by DFT calculations [25,26,27]. PtCu SAA catalyst exhibited high selectivity, stability and resistance to poisoning in hydrogenation of butadiene to butenes under industrial conditions.

Analyzing the results of these studies, it can be assumed that high selectivity of single-atom alloy catalysts in alkyne hydrogenation can be attributed to a number of their structural characteristics. (1) The single-atom surface structure ensures structural homogeneity of the active centers and identical adsorption and catalytic characteristics. (2) Modification of Pd catalysts with a second metal prevents formation of palladium hydride, which presence leads to over-hydrogenation to the alkane [9,28]. (3) Absence of multiatomic surface Pd_n_ centers (where *n* ≥ 2) and the occurrence of hydrogenation exclusively on Pd_1_ sites have a significant effect on the overall reaction mechanism. In particular, it facilitates the alkene desorption from the catalyst surface and prevents its further hydrogenation.

From the viewpoint of the hydrogenation mechanism, the fundamental difference between traditional Pd and SAA catalysts is that the reaction on SAA surface occurs on active centers comprising a single atom of Pd (Pd_1_) surrounded by essentially inactive Ag atoms. Taking into account that hydrogenation of various organic compounds typically proceeds via a bimolecular mechanism, which for the current case implies that adsorption and activation of hydrogen and alkyne occur on identical Pd_1_ sites separated from each other by Ag atoms, it can be expected that the reaction mechanism can be significantly different on SAA Pd catalysts compared to traditional Pd catalysts, influencing thereby the reaction kinetics. Unfortunately, up to date, kinetic studies aimed at revealing specific features of the hydrogenation kinetics on single-atom alloy catalysts are scarce. It should be especially noted that systematic kinetic studies of liquid-phase hydrogenation of substituted alkynes on SAA catalysts are practically absent, despite the considerable industrial importance of these reactions.

The scientific significance of this experimental study on kinetic analysis of the liquid-phase alkyne hydrogenation on SAA catalysts with subsequent kinetic modeling is in discrimination of the hydrogenation mechanisms proposed in the literature for SAA catalysts. Moreover, since one of the main advantages of single-atom alloy catalysts is their intrinsically higher selectivity, such investigation also makes it possible to explore in a quantitative way the influence of various factors on selectivity, such as a possible contribution of the competitive adsorption of reagents, direct alkyne-to-alkane hydrogenation, and the ratio of alkyne/alkene adsorption constants.

Our research comprised the following stages:

(1) Analysis of the mechanisms proposed in the literature for the selective hydrogenation of alkynes on SAA catalysts;

(2) Experimental measurements of the traditional kinetic characteristics (reaction orders with respect to alkyne and hydrogen) and study of the effects of the H_2_ partial pressure and 1-phenyl-1-propyne concentration on the selectivity of the process over the entire range of alkyne conversion. Selection of the appropriate reaction mechanism based on experimental data for the following kinetic simulation;

(3) Kinetic modeling of the process on the basis of the selected reaction mechanism and evaluation of the model capability to capture the kinetic regularities and the concentration profiles for PdAg SAA catalyst.

Analysis of the proposed hydrogenation mechanisms shows that several key stages can be most sensitive to the specific structure of the active sites of SAA catalysts thus influencing the hydrogenation kinetics.

One of the important stages of hydrogenation process is the activation of molecular hydrogen via dissociative adsorption on the metal surface. According to the classical viewpoint, for dissociative hydrogen adsorption the active centers of the catalyst must consist of 2 or more active metal atoms [29,30]. However, the results of contemporary studies using theoretical and computational methods evidenced that this stage can occur on a single metal atom. Thus, Tierney at al. [31] confirmed a possibility of molecular hydrogen dissociation on individual isolated Pd atoms on the surface of PdCu single atom alloys. Analogous results were obtained for alloyed single-atom Pd catalysts with Ag and Au as the host metal [32]. The authors demonstrated for a Pd-Ag SAA catalyst that Pd atoms isolated in the Ag matrix are capable to accelerate H_2_ activation. Hydrogen atoms from dissociated H_2_ then spillover to the Ag surface, where they are weakly bound and therefore are easily consumed in acrolein hydrogenation. More recently SAA CuPd_0.006_/SiO_2_ catalysts prepared by the co-impregnation method exhibited high selectivity with respect to ethylene in selective hydrogenation of acetylene [22]. Density functional theory calculations showed that isolation of Pd atoms by Cu and the transfer of electrons from copper to palladium promotes hydrogen dissociation.

Thus, the experimental and theoretical results accumulated to date allow concluding that dissociation of dihydrogen on a Pd single-atom site alloyed to the surface of a host metal is possible and may proceed even easier than on the surface of palladium metal per se. Subsequently, it can be assumed that this stage is not the one determining the overall rate.

Another important step of alkyne hydrogenation is adsorption and activation of alkyne and alkene molecules. The hydrogenation mechanisms discussed in the literature suggest that their adsorption occurs either on isolated atom of the active metal, or on the surface of the host component. Thus, for selective butadiene hydrogenation on Pt-Cu SAA it was suggested that isolated Pt atoms are the active sites for hydrogen adsorption and dissociation, while hydrocarbon molecules adsorb on Cu surface.

At low loadings, Pt exists as individual isolated atoms alloyed into the Cu surface. These single Pt_1_ sites activate dissociation of dihydrogen and spillover of H to Cu. Spillover of H species reacting with butadiene adsorbed on Cu surface eventually lead to butene formation followed by its desorption. Weak binding of hydrogenated intermediates on Cu surface favors high selectivity in butene [24]. A similar mechanism has been proposed for hydrogenation of acetylene and styrene on Pd-Cu SAA catalyst based on theoretical considerations and experimental data [16,18]. It can be assumed that, in this case, hydrogenation kinetics can be described by the classical Langmuir–Hinshelwood mechanism suggesting non-competitive adsorption of hydrogen and hydrocarbon molecules on Pd_1_ and Cu active sites respectively.

However, the mechanism proposed for Pd-Cu SAA is not valid for Pd-Ag and Pd-Au SAA catalysts. According to the current views spillover of hydrogen atoms over the surface of Pd-Ag SAAs is thermodynamically and kinetically unfavorable due to a low binding energy of Ag-H [33,34,35]. Similar conclusions were proposed for AuPd catalytic systems [36,37]. This is confirmed by DFT calculations of the energy of adsorbed H atoms performed by Darby et al. [15]. It was shown that Ag-H energies for Ag (111) surfaces and for Ag (111) surfaces doped with Pd are extremely low, ranging from −0.12 to −0.16 eV [38]. Taking into account that the Ag surface is not capable to adsorb and activate alkynes [39,40] most authors agree that both hydrogen and alkyne adsorption occurs on isolated palladium centers. Moreover, investigation of the surface structure and H_2_ dissociation on PdAg single-atom alloy demonstrated that for successful partial hydrogenation of acetylene it is necessary that isolated Pd sites, on which the adsorption of hydrogen and alkyne molecules takes place, should be close enough to each other [35].

Analyzing the proposed reaction pathway from the viewpoint of the hydrogenation kinetics, it should be mentioned, that adsorption of both hydrogen and alkyne molecules occurs on the same type of active sites (single-atom Pd_1_ sites). Therefore, it can be assumed that a kinetic model for hydrogenation should take into account competitive adsorption of the reagents to provide an adequate and physically reasonable description of the process.

On the other hand, an alternative mechanism, which does not require hydrogen spillover to the Ag surface, has been proposed by Liu [41]. The author studied the mechanism of acetylene hydrogenation on the surface of a single-atom PdAg catalyst using the density functional theory. According to the calculations, one of the variants of the hydrogenation mechanism suggests co-adsorption of acetylene and molecular hydrogen on the same Pd atom, followed by dissociation of H_2_ due to a low barrier for this reaction (E_rb_ = 0.05 eV). Dissociation is followed by a sequential addition of hydrogen atoms to acetylene with the consecutive formation of C_2_H_3_ and C_2_H_4_, which is desorbed from the Pd_1_ site. If hydrogenation proceeds according to the proposed mechanism, one can expect that its kinetic model may be similar to the kinetic description of the reaction, which assumes non-competitive adsorption.

In this study the focus was on studying experimentally hydrogenation of a substituted alkyne on a Pd-Ag SAA catalyst and analyzing its kinetics with eventual kinetic modeling. The choice of the system was dictated by the fact that Pd-Ag catalysts are among the most effective industrial systems for the removal of acetylene impurities from ethylene, and that excellent selectivity of PdAg SAA was previously reported in liquid-phase [12] and gas-phase alkyne hydrogenation [20,42,43,44,45,46]. Thus, in hydrogenation of diphenylacetylene excellent (ca. 98%) selectivity toward the desired alkene was observed at DPA conversion > 95%.

This work is a continuation of the previous studies of the authors on the kinetics of substituted alkynes hydrogenation on SAA Pd-Ag catalysts. In particular, hydrogenation of an asymmetric 1-phenyl-1-propyne was investigated from a practical point of view since selective hydrogenation of unsymmetrical alkynes is of considerable interest. Our research was aimed at developing a kinetic model of the process based on the reaction mechanism proposed in the literature and capable of adequately describing the experimental data for the PdAg SAA catalyst.

## 2. Materials and Methods

### 2.1. Materials

1-phenyl-1-propyne (98%, Sigma-Aldrich Chemie GmbH, Taufkirchen, Germany) and n-hexane (98%, Merck KGaA, Darmstadt, Germany) were used as a substrate and a solvent, respectively, after additional purification by distillation under Ar atmosphere. Alumina powder (98.9%, SBET = 56 m^2^/g, PURALOX 200/55, Sasol, Germany) was used as a carrier. As catalyst precursors Pd(NO_3_)_2_ and Ag(NO_3_)_2_ were used (Sigma-Aldrich, ≥99.0%). For the catalyst pre-treatment and catalytic tests the following gases and gas mixtures were applied: 5 vol.% H_2_/Ar (JSC Moscow Gas Processing Plant (MGPP), Vidnoe, Russia), H_2_ (99.9999%, MGPP), He (99.999%, MGPP), N_2_ (99.999%, MGPP), Ar (99.9999%, MGPP), 0.5 vol.% CO/He (Linde Gas Rus, Balashikha, Russia).

### 2.2. Catalyst Preparation

Both Pd_1_Ag_3_/Al_2_O_3_ and Pd/Al_2_O_3_ catalysts were synthesized using the incipient-wetness co-impregnation.

**Scheme 1:**
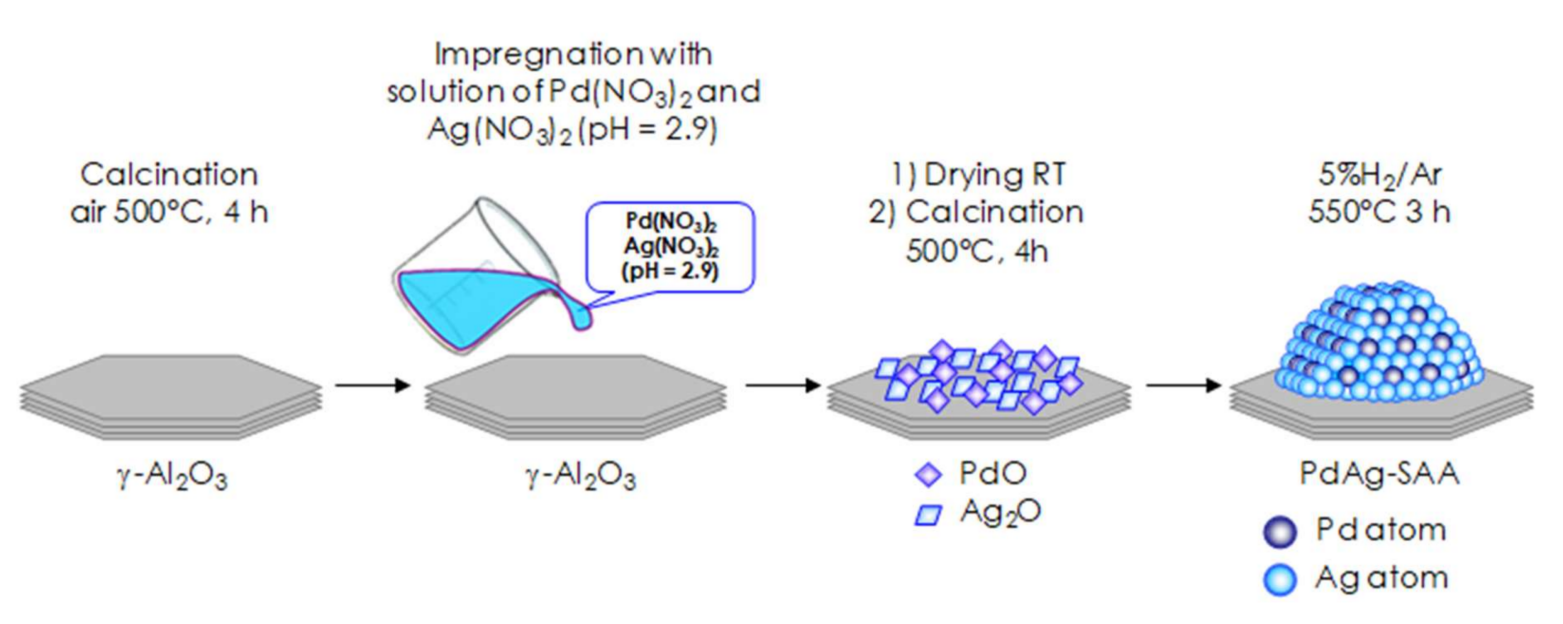
Preparation of Pd_1_Ag_3_/Al_2_O_3_ catalyst via impregnation.

Scheme 1 summarizes the catalyst preparation steps. For Pd_1_Ag_3_/Al_2_O_3_ preparation alumina powder pre-calcined in dry-air at 500 °C for 4 h was impregnated with the aqueous solution containing both Pd(NO_3_)_2_ and Ag(NO_3_)_2_ which was acidified by diluted HNO_3_ to pH of 2.9 to avoid precipitation of Pd or Ag hydroxides. The impregnated material was dried overnight at room temperature, then calcined in dry air at 500 °C for 4 h followed by reduction reduced in 5%H_2_/Ar at 550 °C for 3 h. The material was cooled down in 5%H_2_/Ar to 200 °C, and after that the gas was switched to N_2_ (99.999%) and the sample was cooled down to room temperature. The selection of reduction temperature was based on our previously reported data [10]. It was shown that this temperature is sufficient for the reduction of metallic components providing a required degree of Pd and Ag atoms mobility and formation of uniformly distributed Pd–Ag nanoparticles.

The weight loading of both metallic components on the supported catalysts were determined via inductively coupled plasma atomic emission spectrometry (ICP-AES). The data obtained showed the metal content of 2.1 wt.% Pd and 5.99 wt.% Ag.

As a reference 0.5 wt. % Pd/Al_2_O_3_ catalyst prepared in a similar way was used. To ensure a similar size of metal particles in both Pd_1_Ag_3_/Al_2_O_3_ and Pd/Al_2_O_3_ catalysts, the monometallic Pd sample was reduced in 5%H_2_/Ar at 700 °C for 3 h. The same preparation technique was used for synthesis of Ag/Al_2_O_3_ catalyst used for XPS study. Pd_1_Ag_3_/Al_2_O_3_, Pd/Al_2_O_3_ and Ag/Al_2_O_3_ catalysts were designated as Pd_1_Ag_3_, Pd, and Ag respectively.

### 2.3. Catalyst Characterization

Transmission Electron Microscopy (TEM). To collect TEM micrographs a Hitachi HT 7700 electron microscope (Japan) was used at an accelerating voltage of 100 kV in the bright-field regime. For TEM analysis the catalysts were finely crushed and ultrasonically dispersed to a copper gauze (d = 3 mm) from an isopropanol suspension. The gauze was covered with a carbon film for a better contrast [47]. More than 180 metal particles located in different parts of the samples were measured to estimate the particle size distribution. The average metal particle size (*d*_av_) was calculated according to the following formula:

*d*_av_ = Σn_i_d_i_/n,

Here n_i_ is the number of nanoparticles with a diameter d_i_; n is the total number of nanoparticles.

BET surface area analysis. Surface areas of parent alumina and supported Pd/Al_2_O_3_ and Pd_1_Ag_3_/Al_2_O_3_ catalysts were measured on a Micrometrics ASAP 2000N instrument using N_2_ adsorption-desorption isotherms and Brunauer-Emmet Teller (BET) techniques. Before the measurements all samples were degassed under vacuum at 200 °C for 4 h. N_2_-BET measurements were performed at liquid nitrogen temperature.

The diffuse reflectance IR spectroscopy of adsorbed CO (DRIFTS-CO). Spectra were collected with a Tensor 27 IR spectrometer (Bruker, Germany). The instrument was equipped with a Harrick Diffuse Reflectance Kit (Harrick Scientific Products, UK) for in situ measurements in a flow of different gases. 20 mg of the reduced catalyst was placed in a thermostatically controlled cell with CaF_2_ windows, heated in flowing Ar (30 mL/min) to 500 °C for 1 h, and in reduced flowing 5% H_2_/Ar (30 mL/min) at 550 °C for 1 h. After that the sample was cooled first to 300 °C in 5% H_2_/Ar (30 mL/min) and then to 50 °C in Ar (30 mL/min) The background spectra were collected at 50 °C in flowing He (30 mL/min). The spectra of adsorbed CO were collected at 50 °C in flowing CO in He (0.5 vol.% CO) for 10 min (30 mL/min; 250 scans; resolution, 4 cm^−1^).

Temperature-programmed Pd hydride decomposition (TPHD). Analysis was performed in an automatic continuous flow setup equipped with a thermal conductivity detector (TCD), a water vapor trap, and a data acquisition and processing units. The pre-reduced catalysts (25 mg) were placed in the reactor and treated in Ar at 300 °C for 1 h. Then the catalysts were treated in a 5% H_2_/Ar flow for 15 min at 550 °C (Pd_1_Ag_3_/Al_2_O_3_) and 700 °C (Pd/Al_2_O_3_). After that catalysts were cooled down to 0 °C and purged with Ar. TPHD analysis was performed in the temperature range from 0 to 300 °C with 10 °C/min ramp and the hydrogen evolution was continuously measured by TCD.

X-ray photoelectron spectroscopy (XPS). X-ray photoelectron spectra were measured on a photoelectron spectrometer SPECS (Germany) using an AlKα source (hν = 1486.6 eV, 150 W). The binding energy (BE) scale was preliminarily calibrated by positions of the peaks of gold and copper core levels: Au*4f*_7/2_—84.0 eV and Cu*2p*_3/2_—932.7 eV. During the measurements, pressure of the residual gases did not exceed 8 × 10^−9^ mbar. Dispersed powder samples of the reduced Pd/Al_2_O_3_ and Pd_1_Ag_3_/Al_2_O_3_ were supported on the stainless steel mesh spot welded on a standard sample holder. Before measurements, the catalysts were re-reduced in a high-pressure cell of spectrometer in hydrogen (*p* = 130 mbar) at 500 °C for 60 min. Thereafter the sample was transferred to the analyzer chamber, and the photoelectron spectra were measured. To estimate the chemical states of the elements on the catalyst surfaces narrow Al*2p*, C*1s*, Pd*3d*, Ag*3d*, and O*1s* regions were measured. Spectral analysis and data processing were performed with an XPS Peak 4.1 program [48]. A linear or Shirley background was added and the peaks were fitted with the Gauss–Lorentz (GL) sum functions. Integrated line intensities were calculated from the area of the corresponding narrow regions (Al*2p*, C*1s*, O*1s*, Pd*3d* and Ag*3d*). The positions of measured spectra were corrected using the C*1s* core level (BE = 284.5 eV) from carbon present in the support. To take into account the surface charging, the Al*2p* line at BE = 74.5 from the support (γ-Al_2_O_3_) were used as an internal standard. The relative content of the elements and the ratio of their atomic concentrations were determined from the integrated intensities of photoelectron lines corrected by their respective atomic sensitivity factors [49].

### 2.4. Catalytic Tests

The liquid-phase hydrogenation was performed in a batch type reactor at 25 °C, P(H_2_) of 5 bar, and 1000 rpm stirring, using *n*-hexane as a solvent [12,13]. The effect of solvent on various catalytic reactions has been studied thoroughly in the literature [50,51,52,53,54,55]. The solvents can exhibit interactions with the substrate and the catalyst, being for example strongly adsorbed on the surface or can influence the solubility of gases in the liquids and other mass transfer aspects. In order to avoid interference of a solvent in the current work a hydrocarbon devoid of any functional groups or heteroatoms was therefore selected.

The stirring rate was selected on the basis of our early study, which showed that external mass-transfer limitations can be avoided at a stirring rate > 600 rpm [56]. Experimental details can be found elsewhere [12,13].

The catalyst was crushed to a fine powder (<10 μm) in order to minimize the internal mass-transfer limitations as proposed by Chaudhari [57]. Reaction products were analyzed by gas chromatography with a Crystal 5000 (Chromatek, Russia) using HP5-MS column of 30 m × 0.25 mm with I.D., 0.25 μm film thickness. The GC was equipped with a flame-ionization detector.

Elucidation of the impact of mass transfer was done by calculating the Weisz-Prater criterion [58]. A low value below 0.02 indicates that the internal mass transfer is not limiting the overall rate. Moreover, limitations by the gas-liquid mass transfer were excluded by experiments with different catalyst loading in the reactor.

The hydrogenation rate *r* (mmol H_2_ g_cat_ ^−1^ min^−1^) was measured on the basis of the rate of H_2_ consumption and on the basis of the GC analysis data as a function of the reaction time. Both methods provided consistent results. The initial hydrogenation rate was determined at low 1-Ph-1-Pr conversion (<30%).

In order to investigate the catalyst stability five catalytic cycles were performed for both Pd/Al_2_O_3_ and Pd_1_Ag_3_/Al_2_O_3_. After each cycle, the catalysts were recovered from the reaction mixture by centrifugation (10,000 rpm, 10 min), washed with *n*-hexane to ensure removal of the product from the catalyst surface, and then dried overnight. Each cycle of hydrogenation was performed with the fresh reactants under the same conditions (see Section 2.4 for details).

After the fifth hydrogenation cycle TEM analysis was performed for Pd/Al_2_O_3_ and Pd_1_Ag_3_/Al_2_O_3_ to ensure that the catalysts retain their structure. The samples were recovered from the reaction mixture as mentioned above. For details of TEM analysis see Section 2.3.

## 3. Results and Discussion

### 3.1. Catalyst Characterization

#### 3.1.1. Temperature-Programmed Pd Hydride Decomposition

Selectivity decline in alkyne hydrogenation over Pd catalysts is frequently attributed to over-hydrogenation by hydrogen from palladium hydride phases [59]. During hydrogenation, hydrogen migrates from the PdHx to the Pd surface, which leads to complete hydrogenation of the adsorbed substrate. As a result, a decrease in the selectivity towards the desired alkene is observed. Modification of the Pd catalyst with a second metal inhibits formation of PdHx. Thus in alkyne hydrogenation the high selectivity of bimetallic catalysts is frequently associated with suppression of overhydrogenation, which is provoked by hydrogen from PdHx [60,61].

**Figure 1:**
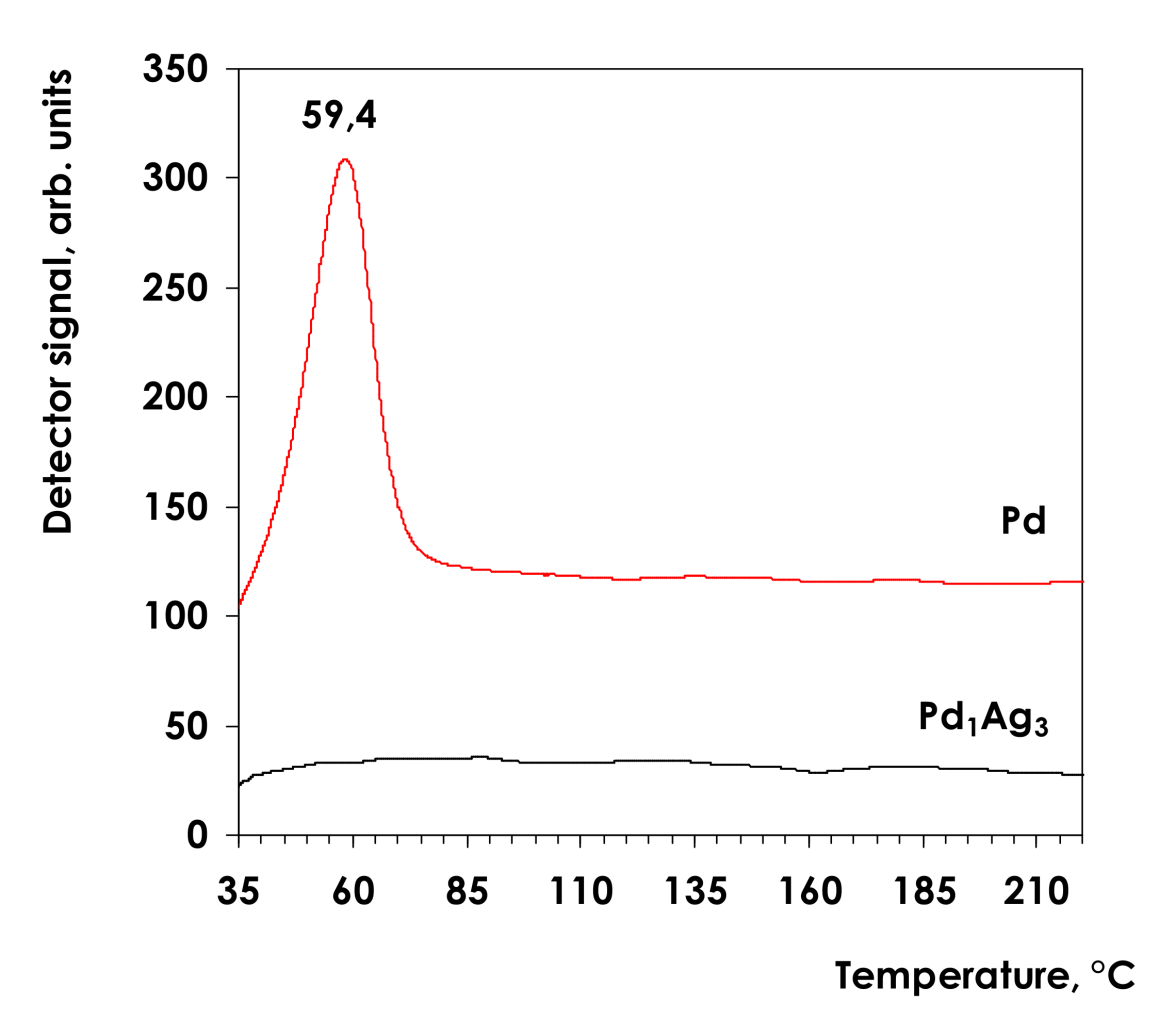
Temperature-programmed Pd hydride decomposition profiles for the monometallic Pd and Pd_1_Ag_3_ SAA catalysts.

To study formation of PdH phases for Pd_1_Ag_3_ and the reference Pd catalysts the samples were saturated with H_2_ followed by TPHD (Figure 1).

For a monometallic Pd catalyst the peak of hydride decomposition is evidently observed with a maximum at ca. 60 °C. This result agrees well with the previously reported data [60,62,63,64]. The calculated H/Pd ratio is 0.30. This value can be explained by a relatively low H_2_ partial pressure upon saturation since the hydrogen content in the H_2_/Ar mixture was only 5%. In contrast, no noticeable peaks of H_2_ evolution were detected in TPHD profiles of the Pd_1_Ag_3_ catalyst indicating inhibition of PdHx formation due to Pd alloying with Ag in bimetallic nanoparticles.

#### 3.1.2. Transmission Electron Microscopy

**Figure 2:**
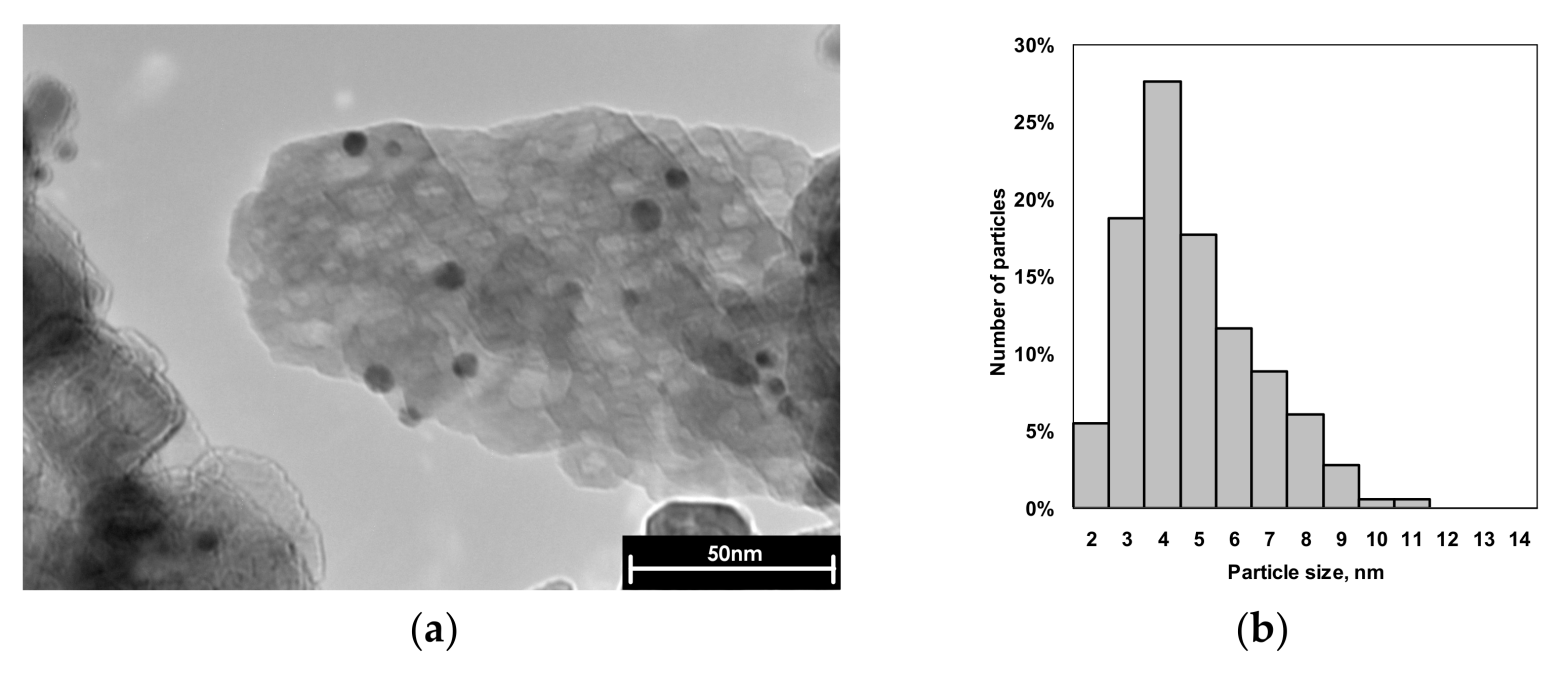

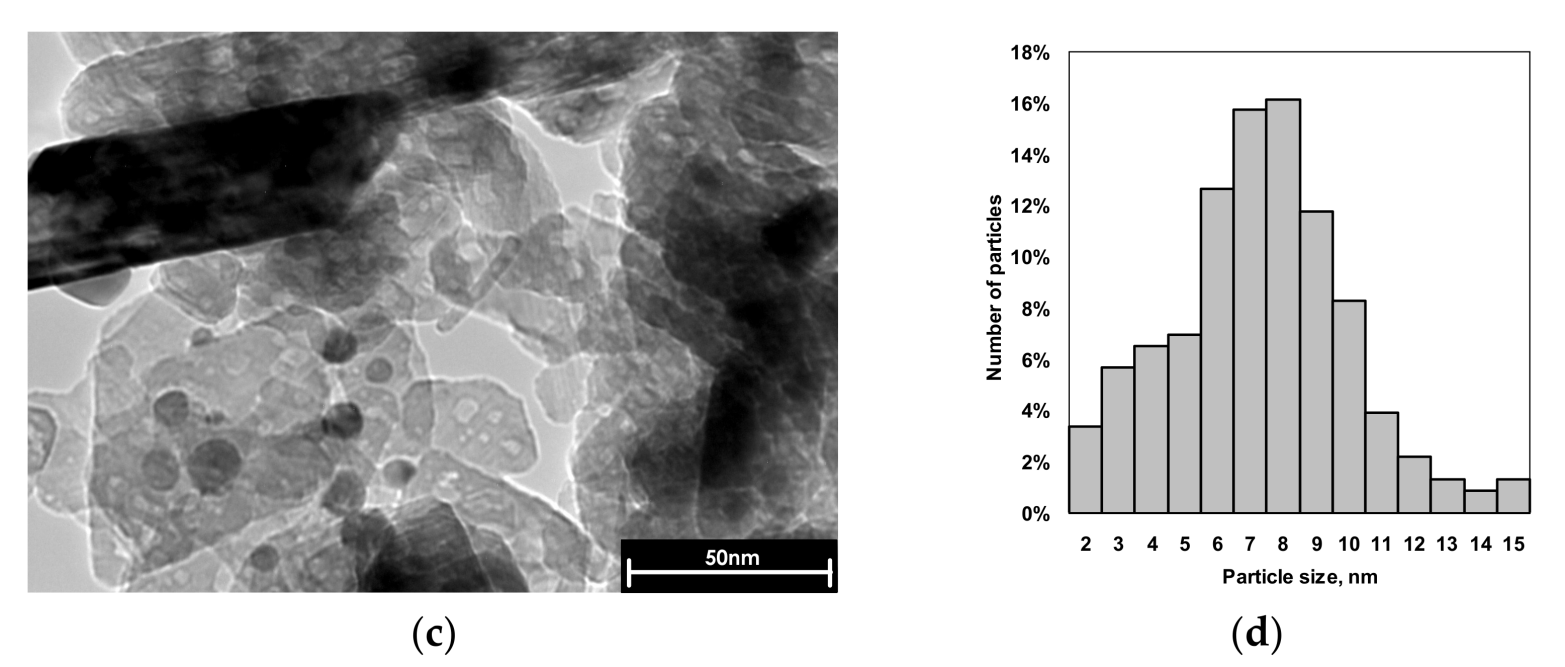
Representative TEM micrographs and particle size distribution of Pd (**a**,**b**) and Pd_1_Ag_3_ (**c**,**d**) catalysts. Average size of particles is 4.3 nm for Pd and 7.0 nm for Pd_1_Ag_3_.

Transmission electron microscopy micrographs are shown in Figure 2. This method was used to estimate the particle size distribution and morphology of supported mono- and bimetallic nanoparticles. Immediately before TEM measurements both samples were re-reduced in a 5%H_2_/Ar flow at 550 °C for 1 h.

As can be seen from Figure 2, Pd and Pd_1_Ag_3_ catalysts contain mostly spherical particles randomly distributed on the surface of carrier with *d*_av_ of 4.4 nm (for Pd) and 7 nm (for Pd_1_Ag_3_). According to previously reported XRD data the bimetallic Pd_1_Ag_3_ particles have a core-shell solid solution structure with the Ag-enriched shell [10].

#### 3.1.3. BET Surface Area Analysis

The BET data are summarized in Table 1. The measurements showed that BET surface areas decreased slightly after Pd loading indicating that the metal component was deposited on the alumina support. After catalytic tests S_BET_ was not essentially altered suggesting that the textural properties did not change under experimental conditions.

#### 3.1.4. DRIFTS-CO

**Figure 3:**
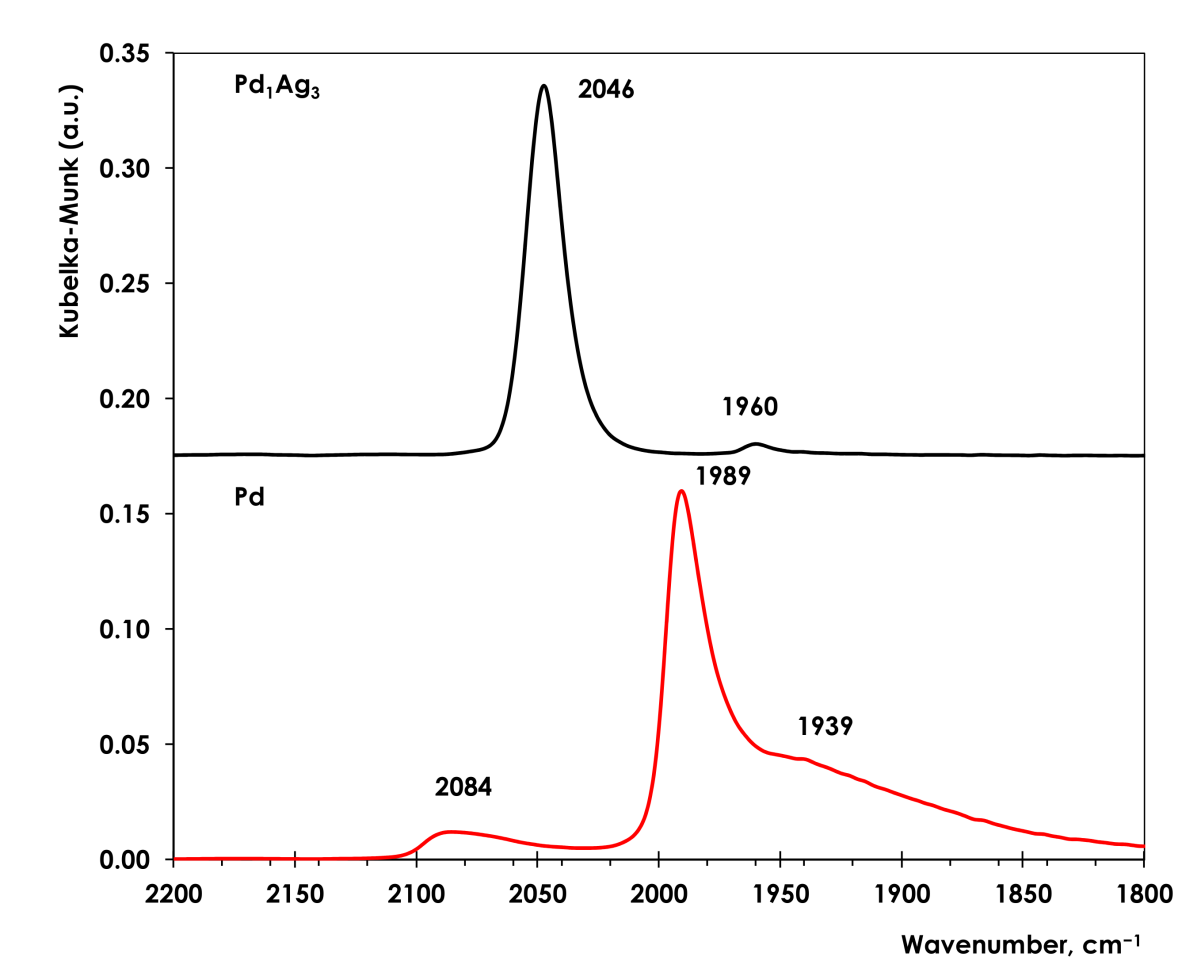
DRIFTS spectra of adsorbed CO on Pd and Pd_1_Ag_3_ catalysts. The spectra were collected at 50 °C in 0.5 vol.% CO/He flow (30 mL/min).

Figure 3 depicts DRIFTS spectra of CO adsorbed on the surface of the reference Pd/Al_2_O_3_ and the SAA Pd_1_Ag_3_ catalyst.

The spectrum of Pd/Al_2_O_3_ contains two distinct absorption bands. The band with a maximum at 2086 cm^−1^ is typical for CO adsorbed linearly on Pd(100) facet and edge sites [65]. The intense signal centered at 1989 cm^−1^ belongs to CO bridging two neighboring Pd atoms. A shoulder at 1939 cm^−1^ is characteristic of threefold bridging CO species and also can be ascribed to bridging CO species on Pd(111) facets [66,67]. As it was previously shown, the wide peak at 1989 cm^−1^ is attributable to bridging CO on steps, edges, and Pd(100) facets [65,68].

In contrast to Pd, the intense symmetric absorption band at 2046 cm^−1^ dominates in the spectrum of CO adsorbed on Pd_1_Ag_3_. The band is attributable to CO linearly adsorbed on Pd atoms. A minor peak at 1960 cm^−1^ measured for Pd_1_Ag_3_ catalyst indicates that the bridged or hollow-bonded CO adsorption has disappeared almost completely. According to the literature, this indicates the disappearance of multiatomic Pd_n_ surface sites (*n* ≥ 2) and predominance of Pd_1_ centers surrounded by Ag atoms, which makes impossible multipoint CO adsorption. The same argument was used by Anderson et al. to prove the formation of the isolated Pd_1_ sites and the absence of adjacent Pd atoms in the case of PdCu/Al_2_O_3_ catalyst, because bridging and triple hollow bonded CO were not observed in FTIR-CO spectra [69]. A similar result was demonstrated for Ag_0.975_Pd_0.025_/SiO_2_ catalyst by Pei et al [20].

Furthermore, analyzing the literature, it can be concluded that the symmetrical peak of low-intensity at 1960 cm^−1^ is attributable to bridge-bonded CO adsorbed on two adjacent Pd atoms (Pd_2_ dimers) on the Pd-Ag nanoparticle surface [65,68]. Note that this peak does not show a tail at a lower frequency characteristic of threefold bridged CO species and indicative of multiatomic Pd ensembles Pd_n_ (*n* > 2).

DRIFTS-CO data allow evaluation of a possible Pd-Pd dimers contribution to hydrogenation. It is important to note that this assessment should be done with great caution because the spectra of adsorbed CO were collected at 50 °C and the saturated CO coverage was not achieved. However, even an approximate estimation can be instructive.

To carry out such estimation the ratio of integral intensities of the linear CO (I_linear_) peak at 2046 cm^−1^ and bridging CO peak (I_bridge_) at 1960 cm^−1^ was calculated using the formula

I_int_ = I_bridge_/(I_linear_ + I_bridge_),

giving the value of 0.04. To evaluate correctly the fraction of Pd_2_ dimers the absorption coefficients of linear and bridging CO should be also taken into account. The absorption coefficient of linear CO was found to be ca. 20–25 fold lower than that of bridged CO [69]. Thus, both low I_bridge_/(I_linear_ + I_bridge_) ratio and the low adsorption coefficient of bridged CO allow us to conclude that a contribution of Pd_2_ dimers is essentially negligible (<0.2%).

#### 3.1.5. X-ray Photoelectron Spectroscopy

**Figure 4:**
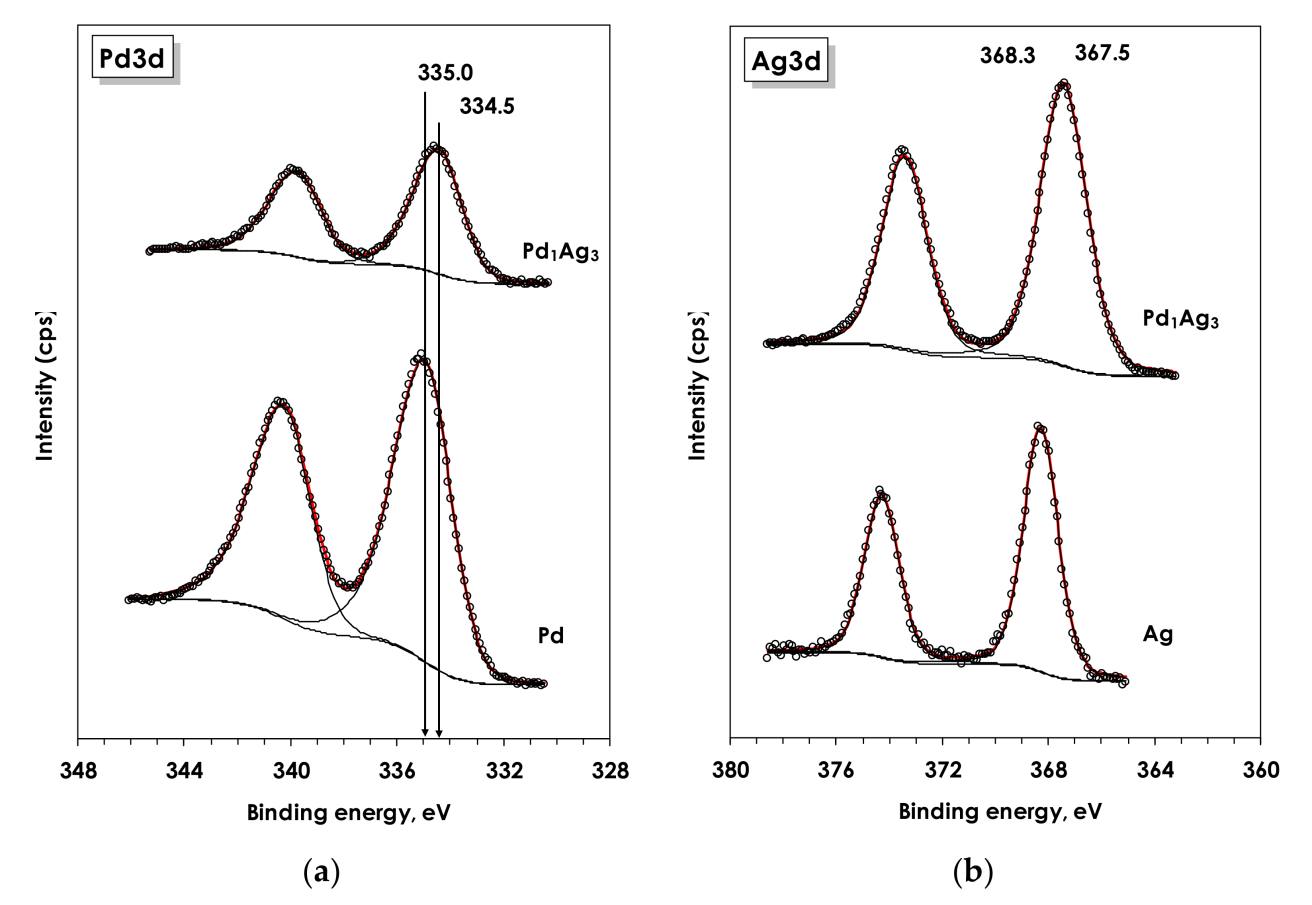
Pd3d (**a**) and Ag3d (**b**) spectra acquired for bimetallic Pd_1_Ag_3_/Al_2_O_3_ and monometallic Pd/Al_2_O_3_ and Ag/Al_2_O_3_.

Figure 4 display the Ag3d and Pd3d spectra acquired for mono- Ag- and Pd and bimetallic PdAg samples reduced in 130 mbar H_2_ at 500 °C. The binding energies (BE) of Pd*3d*_5/2_ and Ag*3d*_5/2_ peaks for the reduced monometallic catalysts are 335.0 and 368.3 eV for Pd and Ag catalysts, respectively. Such values are typical for Pd and Ag in the metallic state [70,71,72]. In the case of the bimetallic sample the BE of Pd*3d* and Ag*3d* shifted to lower values—334.5 and 367.5 eV, respectively. Such shifts are usually assigned to the formation of alloyed PdAg nanoparticles [46,70,71], with the Pd/Ag atomic surface ratio (~0.31) in the current case closely corresponding to the 1:3 stoichiometry of Pd_1_Ag_3_ bimetallic particles.

### 3.2. Catalytic Performance of Pd/Al_2_O_3_ and Pd_1_Ag_3_/Al_2_O_3_ Catalysts in Alkyne Hydrogenation

#### 3.2.1. Comparison of Kinetic Profiles

**Figure 5:**
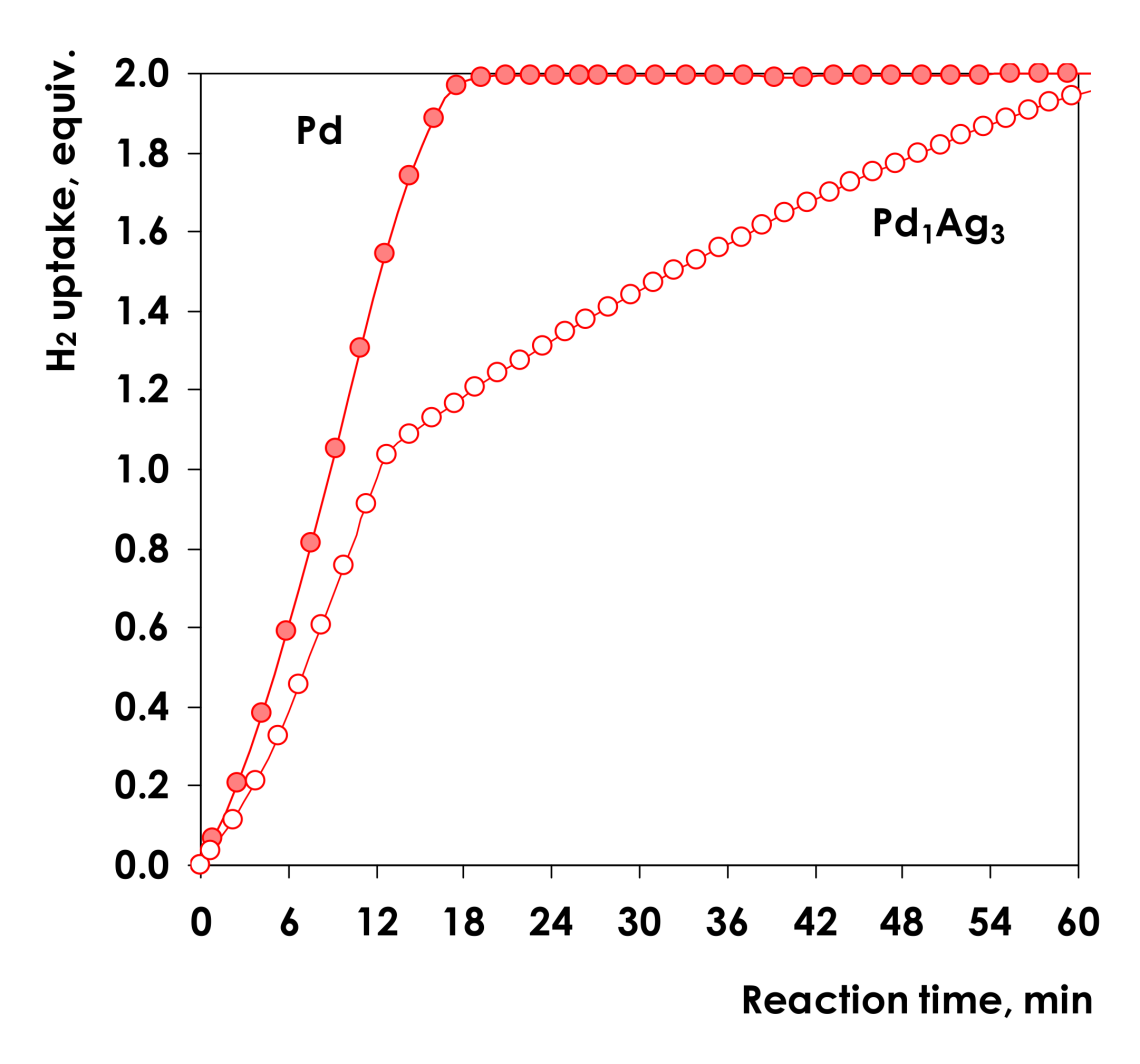
Hydrogen uptake vs. reaction time in the liquid-phase hydrogenation of 1-Ph-1-Pr over Pd and Pd_1_Ag_3_. Conditions: T = 25 °C, P(H_2_) = 5 bar, m(Pd) = 1.5 mg, m(Pd_1_Ag_3_) = 7.5 mg, V_C6H14_ = 6 mL.

Typical kinetic profiles of hydrogen uptake in 1-Ph-1-Pr hydrogenation on Pd and Pd_1_Ag_3_ samples are displayed in Figure 5.

Note that all catalysts were additionally reduced (5%H_2_/Ar flow, 550 °C, 1 h) immediately before catalytic tests. According to previously reported data, this temperature is sufficient to completely reduce PdOx oxide species and to obtain nanoparticles of PdAg solid solution [10].

The kinetic profile for the conventional supported Pd catalyst shows a continuous uptake of hydrogen up to 2 equivalents, corresponding to complete alkyne to alkane conversion. The reaction rate calculated on the basis of the hydrogen uptake rate below 1 hydrogen equivalent (alkyne to alkene hydrogenation, r_1_), is 70.7 mmol H_2_∙g_cat_^−1^∙min^−1^. After the uptake of 1 equivalent of hydrogen, the reaction rate practically does not change and is equal to 80.6 mmol H_2_∙g cat^−1^∙min^−1^. The obtained data show that the rate of hydrogenation does not practically change or even slightly increases after completion of alkyne hydrogenation.

In contrast to Pd catalyst, the kinetic profile of Pd_1_Ag_3_ catalyst exhibits a pronounced decrease in the slope after the uptake of one H_2_ equivalent clearly indicating a decrease in the hydrogenation rate after completing alkyne hydrogenation. The reaction rate decreases almost 4-fold: from 10.8 mmol H_2_∙g_cat_^−1^∙min^−1^ to 2.7 mmol H_2_∙g_cat_^−1^∙min^−1^ allowing to conclude, that on Pd_1_Ag_3_ catalyst the rate of alkene hydrogenation is significantly lower, than the rate of the alkyne hydrogenation. Note that different amounts of catalysts were used in the experiments with SAA and the monometallic supported catalysts, which explain apparent similarities in the slopes of the kinetic curves plotted vs. reaction time. Results presented in Figure 5 allow one to conclude that on Pd_1_Ag_3_ catalyst the rate of alkene hydrogenation is significantly lower, than the rate of the alkyne hydrogenation. Such decrease in the hydrogenation rate on the second stage of alkene to alkane hydrogenation significantly facilitates the kinetic control of the process, as hydrogenation can be stopped after completion of the alkyne to alkene conversion, thereby preventing the loss of the latter in undesired subsequent hydrogenation to the alkane [72,73].

**Figure 6:**
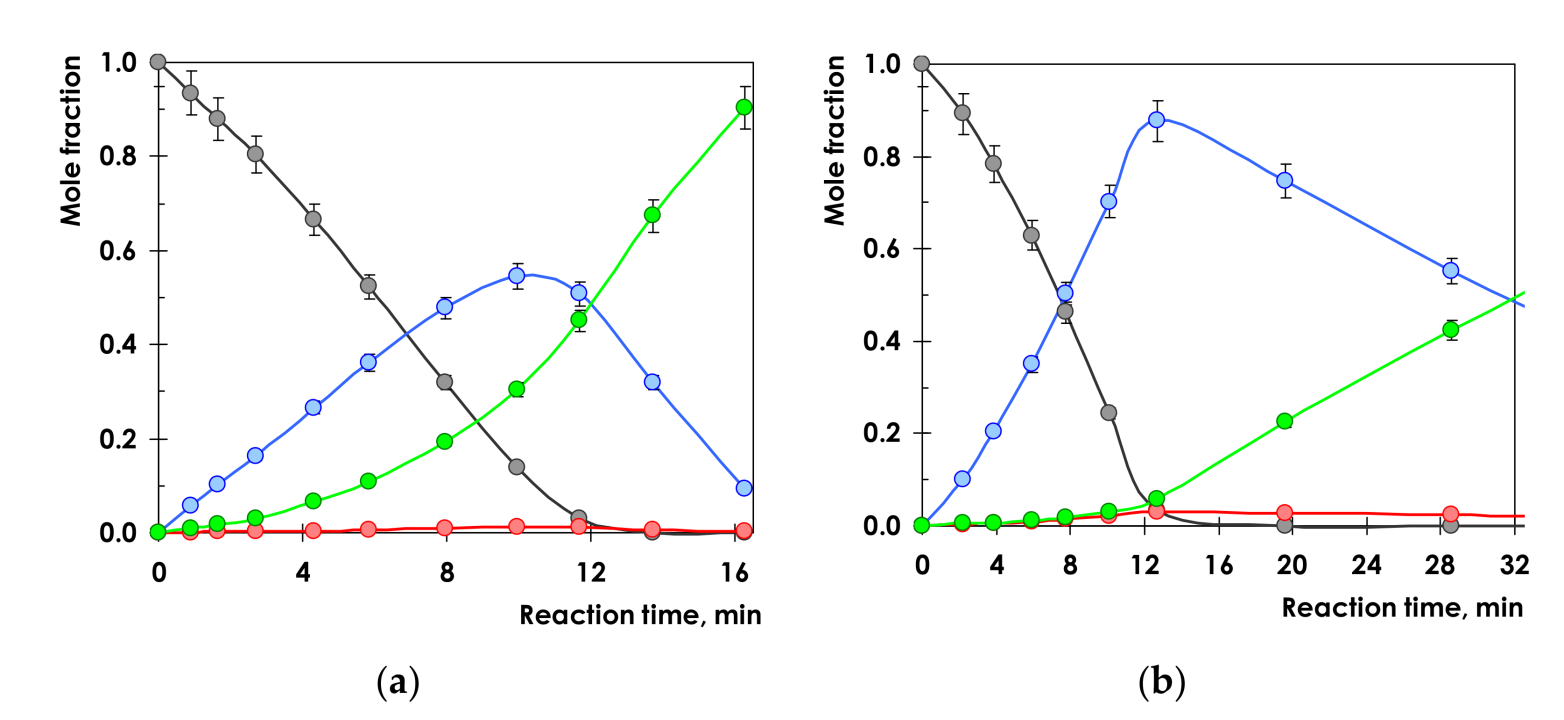
Product distribution for Pd/Al_2_O_3_ (**a**) and Pd_1_Ag_3_/Al_2_O_3_ (**b**) catalysts. Conditions: T = 25 °C, P(H_2_) = 5 bar, m(Pd) = 1.5 mg, m(Pd_1_Ag_3_) = 7.5 mg, V_C6H14_ = 6 mL. The error bars are ±1 standard deviation. Black circles—1-phenyl-1-propyne, blue circles—cis-propenylbenzene, red circles—trans—propenylbenzene; green circles—propylbenzene.

It is informative to compare the dependencies of the reaction product composition on the reaction time. Typical concentration profiles for 1-Ph-1-Pr hydrogenation on Pd and Pd_1_Ag_3_ catalysts are displayed in Figure 6a,b, respectively.

Propenylbenzenes and propylbenzene are among the only products detected. Over both catalysts the reaction proceeds via a sequential reaction network as evidenced by the volcano-type curves for *cis*- and *trans*-alkene. However, the catalyst performances are significantly different. Thus, on monometallic Pd catalyst propylbenzene is detected in the reaction products at the very beginning of the reaction, when 1-Ph-1-Pr conversion is only ca. 7%. At 100% conversion of 1-Ph-1-Pr the content of the undesired propylbenzene exceeds 40% (Figure 6a). In contrast, for Pd_1_Ag_3_ the alkane concentration was found to be below 10% after completion of 1-Ph-1-Pr hydrogenation (Figure 6b). Comparison of the propenylbenzene concentration dependencies on the reaction time shows that the maximum yield of propenylbenzene for the monometallic Pd catalyst does not exceed 54%, while the Pd_1_Ag_3_ SAA it reaches 89%.

The catalyst stability is of significant importance for heterogeneous catalysts determining the lifetime being thus a crucial characteristic in terms of the process economics. Catalyst deactivation stems from poisoning of the active sites, coking, leaching or sintering. The current five hydrogenation cycles were performed to evaluate the stability of both Pd and Pd_1_Ag_3_ catalysts. Details of this experiment can be found in Section 2.4. Figure S1 displays concentration dependencies for mono- and bimetallic catalysts during the fifth hydrogenation cycle. It can be clearly seen that the performance of fresh and recycled catalysts is essentially identical without any noticeable changes in activity or product distribution. These data suggesting absence of catalyst deactivation agree well with the TEM analysis of catalysts after the fifth catalytic tests (see Figure S2) as well as with BET results (see Table 1).

#### 3.2.2. Effect of the Substrate Concentration

**Figure 7:**
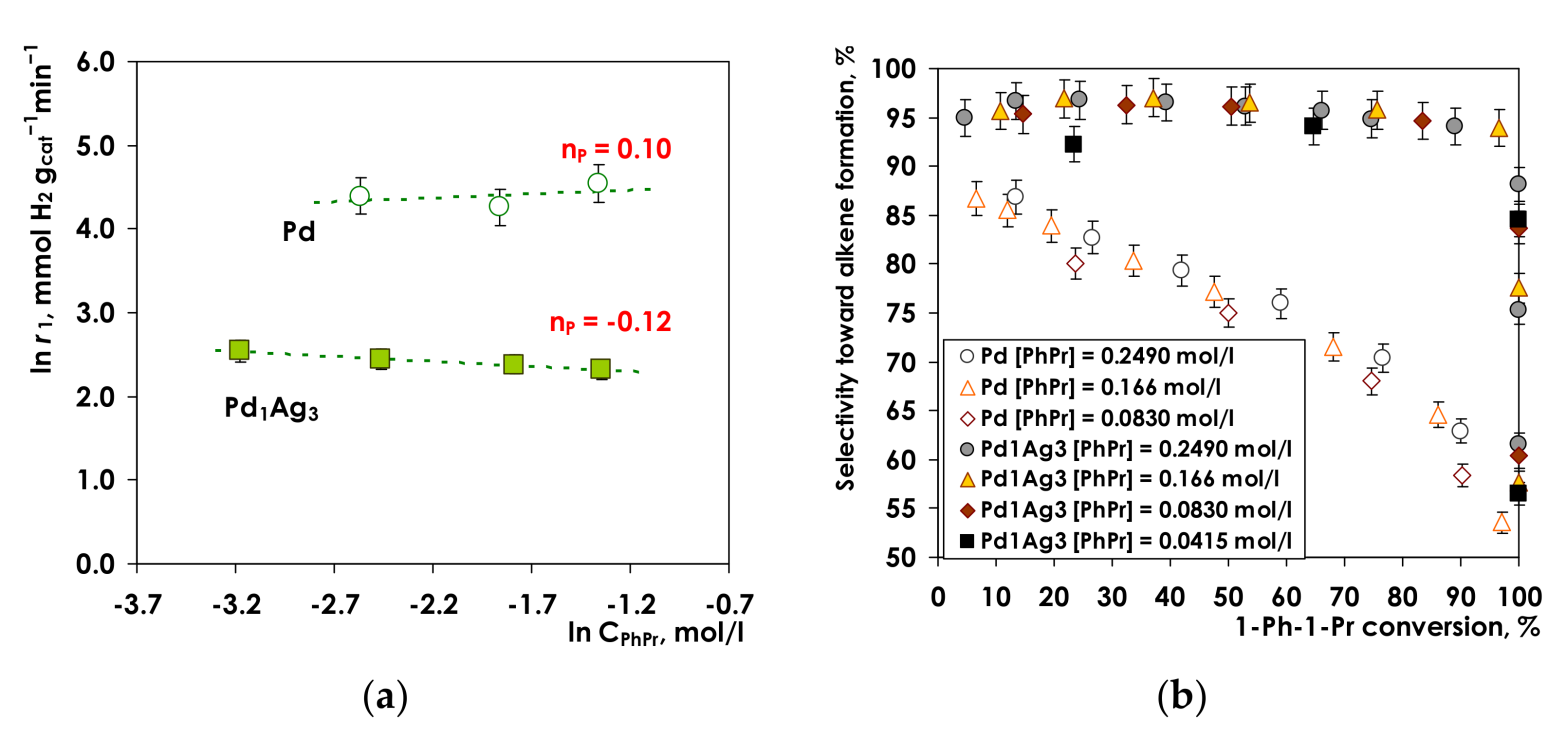
Hydrogenation of 1-Ph-1-Pr over Pd and Pd_1_Ag_3_: (**a**) Reaction rate vs. the initial 1-Ph-1-Pr concentration; (**b**) Selectivity toward propenylbenzene vs. 1-Ph-1-Pr conversion for various initial 1-Ph-1-Pr concentrations. Conditions: T= 25 °C P(H_2_) = 5 bar, m(Pd) = 1.5 mg, m(Pd_1_Ag_3_) = 7.5 mg, V_C6H14_ = 6 mL. The error bars are ±1 standard deviation.

*Reaction order.* The effect of the 1-Ph-1-Pr concentration on the hydrogenation rate was determined for Pd and Pd_1_Ag_3_ samples at a constant H_2_ pressure of 5 bar. Alkyne concentration was varied in the range of 0.0415–0.249 mol/L (Figure 7).

The obtained data reveal distinctly different dependencies of the reaction rate on alkyne concentration. The reaction rate for monometallic Pd catalysts increases with 1-Ph-1-Pr concentration from 80.9 to 93.8 mmol H_2_ g _cat_^−1^ min^−1^. On the contrary, for Pd_1_Ag_3_ SAA catalyst with an increase in the alkyne concentration the reaction rate decreases from 11.5 to 10.1 mmol H_2_ g _cat_^−1^ min^−1^. The generated data can be linearized with an adequate straight line fits with the reaction orders of 0.10 ± 0.06 for Pd and −0.12 ± 0.04 for Pd_1_Ag_3_.

Relatively low values of the reaction order in alkyne are in good agreement with the previously reported data on hydrogenation of phenylalkynes over Pd catalysts [74,75]. Low reaction order is typically attributed to much stronger alkyne adsorption in comparison with the corresponding alkene and alkane. Thus, comparative calculations of acetylene/ethylene competitive adsorption indicated that the heat of adsorption of alkyne (~1.6 eV) is much higher than that of an alkene (~1.0 eV) [76]. Such strong adsorption leads to a high surface coverage of the alkyne even at its low concentrations. Due to the high surface coverage, the reaction rate only marginally depends on the concentration of alkyne [77,78].

It is of interest to analyze possible reasons for the negative order of the reaction rate with respect to alkyne for a Pd_1_Ag_3_ SAA catalyst. The most plausible reason seems to be a more uniform structure of active sites on the catalyst surface. As shown by DRIFTS-CO data, the predominant type of active sites is Pd_1_ isolated with Ag atoms. Thus, activation of the alkyne and dihydrogen occurs on the same type of active sites resulting in a pronounced competitive adsorption of hydrogen and alkyne on Pd_1_ centers. In this case, an increase in the alkyne concentration leads to a decrease in the amount of adsorbed hydrogen diminishing the overall hydrogenation rate. Such rationalization of the reaction order towards the alkyne is important in development of the kinetic model for 1-Ph-1-Pr concentration over a single atom catalyst.

*Selectivity toward alkene formation*. Analysis of the olefin selectivity dependence on the conversion of 1-Ph-1-Pr for Pd and SAA Pd_1_Ag_3_ samples (Figure 7b) demonstrates that the shapes for the selectivity profiles are completely different for these catalysts. For the monometallic Pd an increase in 1-Ph-1-Pr conversion is accompanied with gradual decrease of the alkene selectivity (Figure 7b). It should also be noted that the alkene selectivity tends to decrease at lower alkyne concentration, although this trend is not significant. Thus, at 1-Ph-1Pr conversion of 90%, selectivity decreases from ca. 62% (for [1-Ph-1-Pr] of 0.25 mol/L) to ca. 58% (in the case of [1-Ph-1-Pr] equal to 0.083 mol/L). Previously similar trend was reported for hydrogenation of diphenylacetylene [12], phenylacetylene [79] and 3-methyl-1-pentyn-3-ol [80].

Contrary, for the Pd_1_Ag_3_ catalyst the alkene selectivity of ca. 95–97% remains essentially constant throughout the whole 1-Ph-1-Pr conversion range (Figure 7b). The observed independence of selectivity on alkyne conversion indicates that for the Pd_1_Ag_3_ the contribution of the direct pathway of hydrogenation (1-Ph-1-Pr to the propylbenzene hydrogenation) proceeding simultaneously with selective hydrogenation to the olefin is negligible. One of the plausible explanations is a different strength of alkyne and alkene adsorption on Pd and Pd_1_Ag_3_ SAA catalysts. Alkenes are more strongly adsorbed on monometallic Pd rather than on bimetallic counterpart. The energy of alkene desorption is higher than the activation energy of its hydrogenation; therefore, the hydrogenation of alkene to alkane rather than its desorption from Pd surface becomes an energetically favorable route [81]. Conversely, as it was proposed by Nijhuis et al. [80] and Pei et al. [20], for PdAg_3_ SAA catalyst the single-atom structure of isolated Pd_1_ sites prevents di-σ-bonded alkene adsorption. As a result the π-bonded adsorption occurs, which decreases adsorption energy. In turn, lower alkene adsorption energy facilities alkene desorption hindering its further hydrogenation, which results in high alkene selectivity. Both explanations are mainly focusing on the adsorption strength, however, differences in the rate constants of alkyne vs. alkene hydrogenation should be also considered, as implemented in the kinetic analysis performed in this study.

#### 3.2.3. Hydrogen Pressure Effect

**Figure 8:**
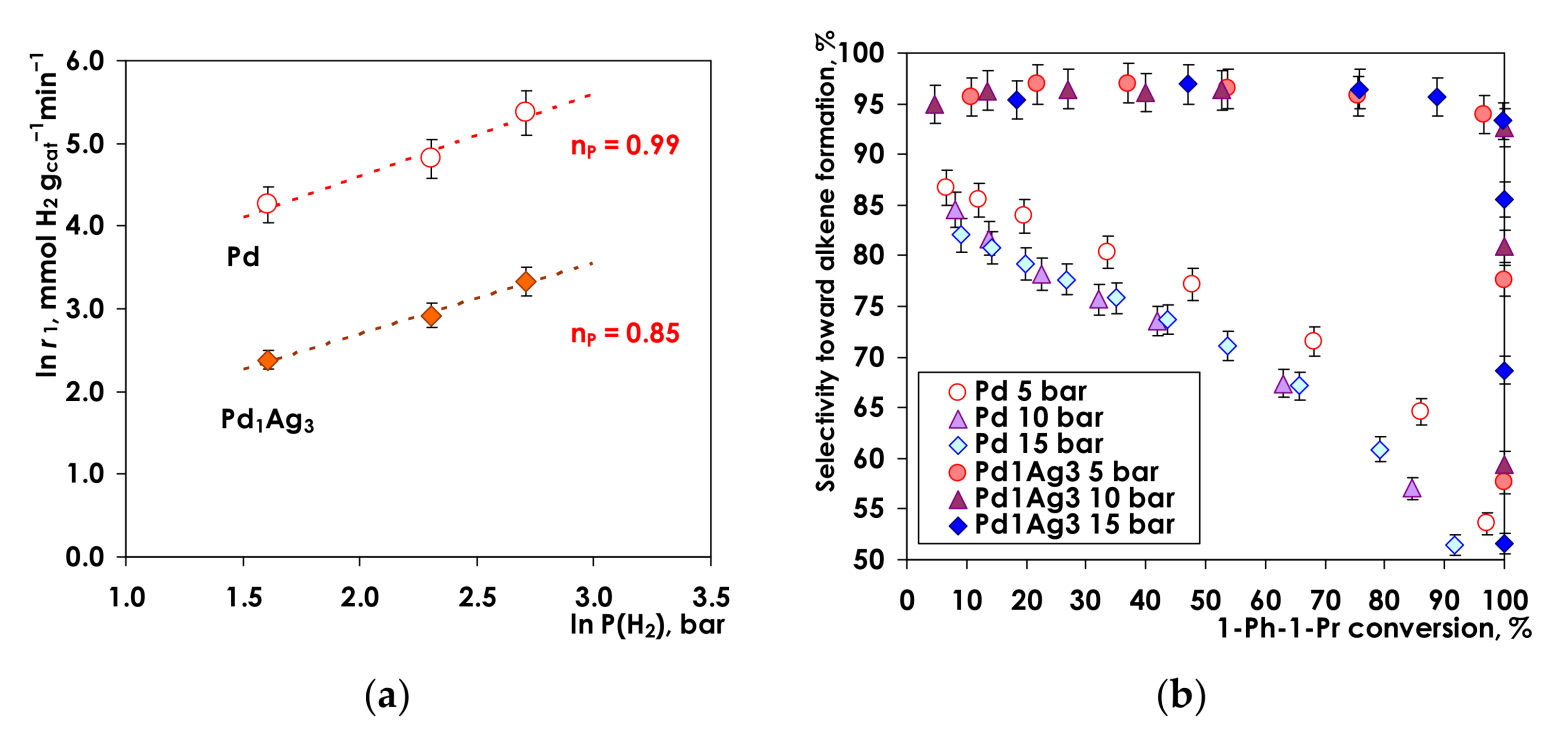
(**a**) Reaction rate vs. hydrogen pressure, (**b**) Selectivity toward alkene vs. 1-Ph-1-Pr conversion at different H_2_ pressures. Conditions: T = 25 °C, V_C6H14_ = 6 mL, m(Pd) = 1.5 mg, m(Pd_1_Ag_3_)= 7.5 mg.

To study the hydrogen pressure effect P(H_2_) was varied from 5 to 15 bar (Figure 8).

The error bars are ±1 standard deviation.

An increase of the initial hydrogenation rates with increasing H_2_ pressure was observed for both catalysts: 3-fold for Pd (from 70.7 to 215.3 mmol H_2_ g_cat_^−1^ min^−1^) and 2.5-fold for Pd_1_Ag_3_ (from 10.8 to 27.9 mmol H_2_ g_cat_^−1^ min^−1^).

The logarithmic plot of the initial reaction rate vs. hydrogen pressure reveals a similar reaction order in hydrogen of 0.99 and 0.85 for Pd and Pd_1_Ag_3_, respectively (Figure 8a). The same trend was earlier demonstrated by several scientific groups in hydrogenation of phenylacetylene [82], acetylene [20], and ethylene [83]. A close to the first order H_2_ pressure dependence suggests that for both catalysts hydrogen is weakly adsorbed on the Pd sites relative to the hydrocarbon species. Similar reaction orders in hydrogen for monometallic Pd and Pd_1_Ag_3_ SAA catalysts suggest that activation of H_2_ occurred predominantly at Pd sites [20]. This conclusion is in line with the DFT calculations of Gonzalez et al. [43] and He et al. [45] who demonstrated that the hydrogen activation is favored on Pd, rather than on Ag sites present in PdAg bimetallic catalysts.

Selectivity to alkene as a function on 1-Ph-1-Pr conversion at different hydrogen pressures is given in Figure 8b for Pd and Pd_1_Ag3. The general shape of selectivity vs. conversion dependencies for both Pd and Pd-Ag catalysts is in a good agreement with the results obtained upon changing the alkyne concentration (Figure 7b).

For Pd_1_Ag_3_ SAA catalyst a high selectivity to propenylbenzene (ca. 95–97%) is essentially independent on alkyne conversion remaining constant within the whole conversion range. “Selectivity-conversion” profiles at different H_2_ pressures are identical indicating that the alkene selectivity is not affected by variation of P(H_2_) from 5 to 15 bars.

For the monometallic Pd catalyst at all hydrogen pressures, a gradual decrease in selectivity from ca. 85 to ca. 55% is observed with an increase in the alkyne conversion. Moreover, a detailed analysis of the selectivity profiles at different H_2_ pressures reveals the evident trend of decreasing selectivity at higher H_2_ pressure. Thus at 85% conversion of 1-Ph-1-Pr selectivity to alkene decreases from ca. 65% to ca. 55% as P(H_2_) increases from 5 bar to 10–15 bar.

Consecutive mechanisms of hydrogenation were discussed in the literature in detail addressing hydrogen pressure dependencies [84,85,86]. When the same dependence of both reaction stages on P(H_2_) holds, selectivity does not depend on P(H_2_) and cannot be explained by a higher surface concentration of adsorbed hydrogen. Alternatively, the low selectivity at elevated pressures stems from the Pd_4_H_3_ hydride formation, as it was shown previously in the C_2_H_2_ hydrogenation over monometallic Pd [87,88].

Thus, a comparative investigation of the hydrogenation kinetics over monometallic Pd and Pd_1_Ag_3_ SAA catalysts revealed several distinct differences specific for single-atom alloy catalyst:

(1) The total hydrogenation rate over Pd_1_Ag_3_ catalyst sharply decreases by a factor of 4 after completion of the 1-Ph-1-Pr hydrogenation, while over monometallic Pd, hydrogenation rate remains almost constant until a complete conversion of the alkyne to propylbenzene.

(2) Selectivity to the alkene for Pd_1_Ag_3_ SAA catalyst is significantly higher than for the monometallic Pd catalyst, remaining essentially constant (95–97%) within the whole range of 1-Ph-1-Pr conversions, while selectivity of the Pd catalyst decreases steadily from ca. 85% to ca. 50–55% as alkyne conversion increases.

(3) Investigation of the 1-Ph-1-Pr concentration effect on the reaction kinetics revealed a negative order in alkyne for Pd_1_Ag_3_ (-0.12 ± 0.01), while for monometallic Pd the reaction order was found to be positive (0.10 ± 0.02). It should also be noted, that there was a significant difference in the effect of initial alkyne concentration on the alkene selectivity for Pd_1_Ag_3_ and Pd catalysts. For the former one selectivity is essentially independent of 1-Ph-1-Pr concentration, whereas for Pd catalyst selectivity tends to decrease at a lower substrate concentration.

(4) Unlike the alkyne concentration effect, the influence of H_2_ pressure on the reaction rate was found to be similar for both catalysts giving close reaction orders in H_2_ for Pd_1_Ag_3_ and Pd catalysts: 0.85 ± 0.05 and 0.99 ± 0.07 respectively. On the other hand, the effect of hydrogen pressure on the selectivity is notably different. For Pd_1_Ag_3_ selectivity did not change upon variation of H_2_ pressure from 5 to 15 bar. On the contrary, on Pd catalyst the selectivity decreases with increasing hydrogen pressure.

### 3.3. Kinetic Modelling

**Figure 9:**
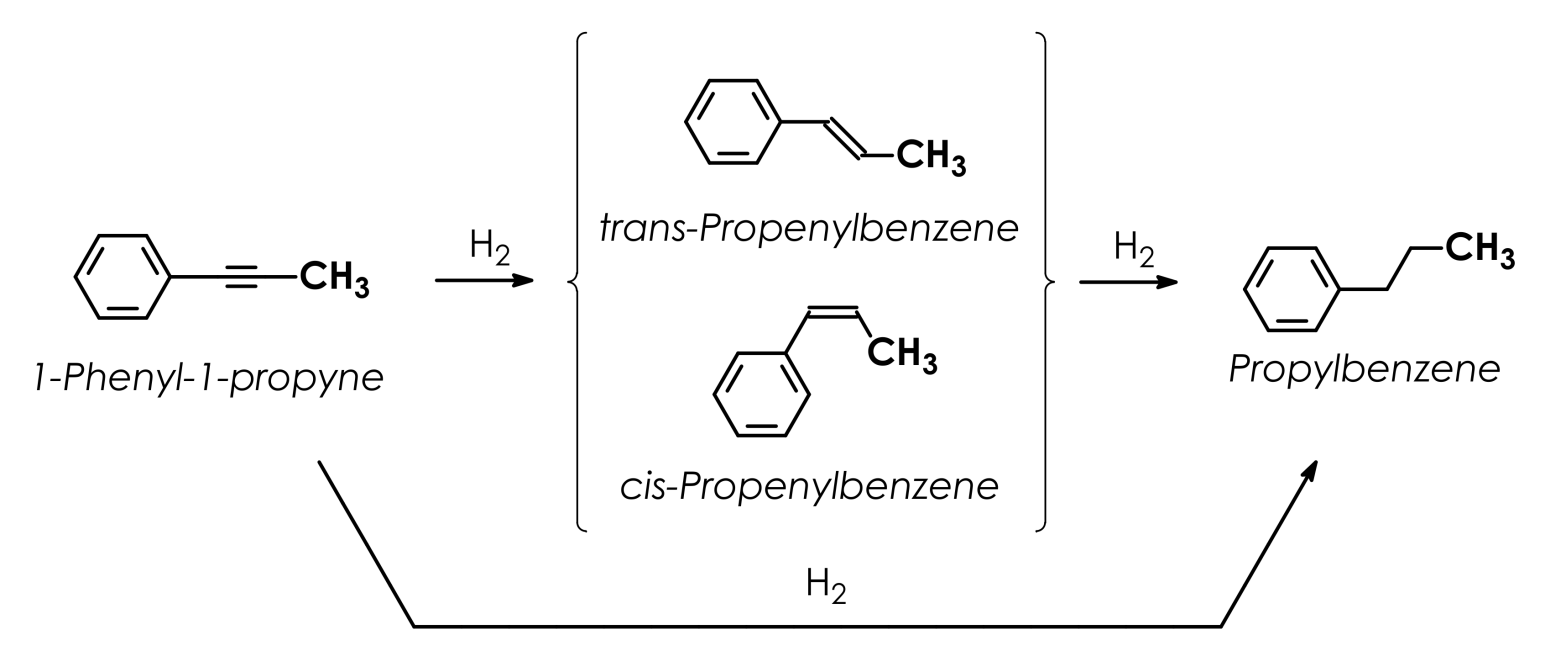
The reaction network in hydrogenation of 1-phenyl-1-propyne.

Specific kinetic behavior of Pd_1_Ag_3_ catalyst can be qualitatively discussed using reaction network described in the previous publications of the authors (Figure 9) [12,13].

According to this scheme, a significant increase in selectivity of Pd_1_Ag_3_ catalyst is attributable to 3 factors:

(1) Negligible contribution of the direct alkyne-to-alkane hydrogenation proceeding simultaneously with the alkyne-to-alkene semi- hydrogenation.

(2) Significant decrease in the rate of alkene hydrogenation compared to the rate of alkyne hydrogenation.

(3) A decrease in the relative adsorption strength of propenylbenzene vs. 1-Ph-1-Pr on SAA Pd_1_Ag_3_ catalysts.

To obtain more insight into the hydrogenation mechanism characteristic of single-atom alloy catalyst on a quantitative basis kinetic modeling was performed using the reaction network proposed earlier and depicted in Figure 9 [12,13].

In order to propose a kinetic model that adequately describes the obtained experimental results, the literature on the kinetics of selective hydrogenation on “single atom” catalysts was analyzed. Although kinetic studies of selective hydrogenation on SAA catalysts are scarce, three possible mechanisms have been proposed.

For Pd-Cu SAA catalysts it was suggested that the reaction proceeds through non-competitive adsorption of the alkyne on Cu surface and H_2_ on Pd_1_ sites followed by dissociation of hydrogen and spillover of hydrogen atoms to the alkyne [16,22]. Unlike Pd-Cu SAA catalyst for Pd-Ag an alternative hydrogenation pathway was proposed, because adsorption of both alkyne or alkene on Ag surface is unfavorable. Therefore, the majority of authors suggested that H_2_ and alkyne/alkene adsorption occurs on isolated Pd1 sites located preferably close to each other [20,35]. For this mechanism, one can expect competitive adsorption of H_2_ and the alkyne, which inevitably affects kinetics of the entire process. Another mechanism of acetylene hydrogenation over PdAg SAA catalyst was developed using the density functional theory, and implies co-adsorption of acetylene and molecular hydrogen on the same Pd atom, followed by dissociation of H_2_, sequential addition of hydrogen atoms to acetylene leading to ethylene formation and its subsequent desorption.

The kinetic regularities observed in the current work (negative reaction order in alkyne, the reaction order of 0.85 in hydrogen, independence of selectivity to the alkene on conversion, hydrogen pressure and the initial alkyne concentration) should be taken into account in proposing a kinetic model consistent with these observations.

In particular, a negative reaction order implies some sort of competition between the alkyne and hydrogen on the same type of sites. This suggestion is in apparent contradiction with ability of an isolated Pd_1_ site to adsorb both reacting molecules simultaneously. At the same time for the SAA catalyst, studied in this work, hydrogen can be adsorbed on a metal Pd site in the vicinity of the site where the alkyne is adsorbed. Subsequently hydrogen diffuses fast over the catalyst surface to the site of alkyne adsorption and reacts with the alkyne. Such mechanistic explanations are in line with the observed kinetic regularities and the current theoretical views regarding adsorption of the alkyne and hydrogen on Pd and Ag.

Subsequently, similar to the gas phase acetylene hydrogenation [89] reporting that selectivity was independent on the initial partial pressure of the substrate, the same type of model comprising competitive adsorption of the reactants (alkyne, the olefin and hydrogen) on the sites of the same type was selected in the current work for kinetic modelling.

For the first reaction route (formation of *cis*-propenylbenzene) the rate ***r^(I)^*** is given by:

(1) $$r^{\left(I\right)}=\frac{k_{1}K_{1P1P}C_{1P1P}K_{H_{2}}P_{H_{2}}}{{\left(1+K_{1P1P}C_{1P1P}+K_{C}C_{C}+K_{T}C_{T}+K_{PB}C_{PB}+K_{H_{2}}P_{H_{2}}\right)}^{2}}$$

where *k*_1_ is the rate constant, *K*_1*P*1*P*_ is the adsorption constant for the reactant, etc., *C*_1*P*1*P*_ is 1-phenyl-1-propyne concentration, etc. In Equation (1) competitive adsorption of hydrogen and organic compounds is assumed on the same type of sites. As adsorption of alkane is typically not as strong as alkynes and alkenes, Equation (1) is simplified to

(2) $$r^{\left(I\right)}=\frac{k_{1}K_{1P1P}C_{1P1P}K_{H_{2}}P_{H_{2}}}{{\left(1+K_{1P1P}C_{1P1P}+K_{C}C_{C}+K_{T}C_{T}+K_{H_{2}}P_{H_{2}}\right)}^{2}}$$

The rate expression for formation of *trans*-propenylbenzene is:

(3) $$r^{\left(II\right)}=\frac{k_{2}K_{1P1P}C_{1P1P}K_{H_{2}}P_{H_{2}}}{{\left(1+K_{1P1P}C_{1P1P}+K_{C}C_{C}+K_{T}C_{T}+K_{H_{2}}P_{H_{2}}\right)}^{2}}$$

While direct hydrogenation of the alkyne to alkane takes the form

(4) $$r^{\left(V\right)}=\frac{k_{5}K_{1P1P}C_{1P1P}K_{H_{2}}P_{H_{2}}}{{\left(1+K_{1P1P}C_{1P1P}+K_{C}C_{C}+K_{T}C_{T}+K_{H_{2}}P_{H_{2}}\right)}^{2}}$$

The expressions for hydrogenation of the *cis* and *trans* olefins are respectively

(5) $$r^{\left(III\right)}=\frac{k_{3}K_{C}C_{C}K_{H_{2}}P_{H_{2}}}{{\left(1+K_{1P1P}C_{1P1P}+K_{C}C_{C}+K_{T}C_{T}+K_{H_{2}}P_{H_{2}}\right)}^{2}}$$

(6) $$r^{\left(IV\right)}=\frac{k_{4}K_{T}C_{T}K_{H_{2}}P_{H_{2}}}{{\left(1+K_{1P1P}C_{1P1P}+K_{C}C_{C}+K_{T}C_{T}+K_{H_{2}}P_{H_{2}}\right)}^{2}}$$

The time (*t*) dependent expressions for concentrations of reactants include catalyst bulk density ρ.

(7) $$\begin{array}{c} -\frac{1}{\rho }\frac{dC_{1P1P}}{dt}=r^{\left(I\right)}+r^{\left(II\right)}+r^{\left(V\right)};\frac{1}{\rho }\frac{dC_{C}}{dt}=r^{\left(I\right)}-r^{\left(III\right)}\\ \frac{1}{\rho }\frac{dC_{T}}{dt}=r^{\left(II\right)}-r^{\left(IV\right)};\frac{1}{\rho }\frac{dC_{PB}}{dt}=r^{\left(III\right)}+r^{\left(IV\right)}+r^{\left(V\right)} \end{array}$$

According to this kinetic model, selectivity towards the intermediate olefin *S_c_* (Equation (8)) is independent of the initial concentration of the alkyne and hydrogen pressure in line with the experimental observations.

(8) $$S_{C}=\frac{r_{C}}{-r_{1P1P}}=\frac{r^{\left(I\right)}-r^{\left(III\right)}}{r^{\left(I\right)}+r^{\left(II\right)}+r^{\left(V\right)}}=\frac{k_{1}}{k_{1}+k_{2}+k_{5}}-\frac{k_{3}K_{C}N_{C}^{}}{(k_{1}+k_{2}+k_{5})K_{1P1P}N_{1P1P}^{}}$$

In Equation (8) *N_c_* is the *cis*-olefin mole fraction. This equation can account also for independence of selectivity to the olefin on conversion when the rate of the olefin hydrogenation is low enough compared to the alkyne hydrogenation or in other words the ratio *k_3_K_C_/(k_1_ + k_2_ + k_5_)K*_1*P*1*P*_ is low.

The model, presented above, was utilized for describing the experimental data gathered with PdAg_3_ SAA catalyst. To assess the quality of the fit the degree of explanation was used, which relates the squared difference in the observed and calculated values with the squared difference between the observed concentrations and the mean values of the calculated ones [90]. Concentration dependences for all reactants at all operation conditions (initial concentrations and pressure) were treated together using the optimization software ModEst [90].

**Figure 10:**
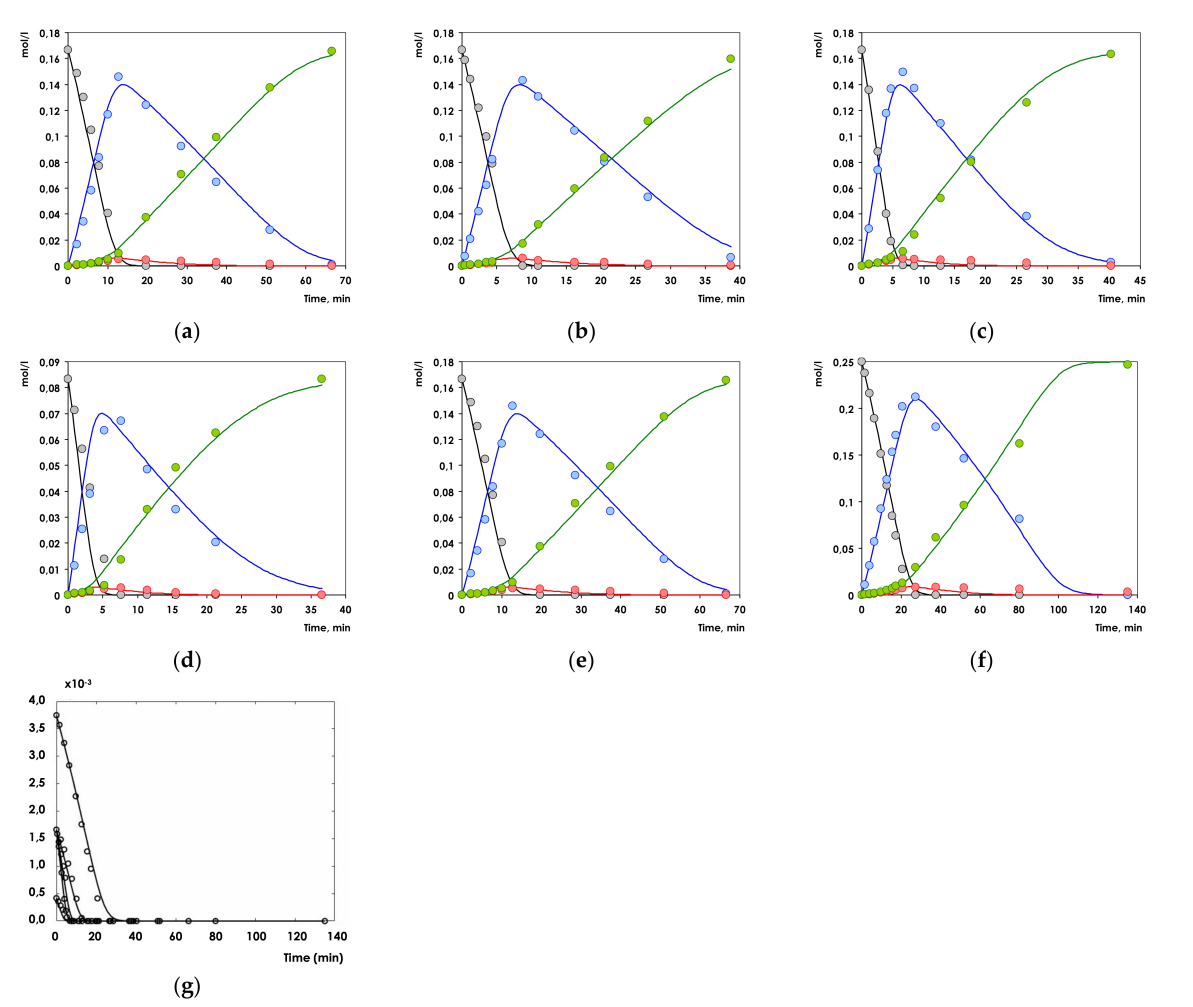
Comparison between calculated (lines) and experimental (symbols) data in 1-Ph-1-Pr hydrogenation over Pd_1_Ag_3_ SAA catalyst under different pressures ((**a**)—5 bar, (**b**)—10 bar, (**c**)—15 bar; C_PhPr_ = 0.166 mol/L) and initial alkyne concentrations ((**d**)—0.0830 mol/L, (**e**)—0.166 mol/L, (**f**)—0.249 mol/L; P = 5 bar). Inlet (**g**) illustrates that the model is capable of adequately capturing the dependence of hydrogenation rate on the initial substrate concentration. Black circles—1-phenyl-1-propyne, blue circles—*cis*-propenylbenzene, red circles—*trans*- propenylbenzene, green circles—propylbenzene.

Comparison between the experimental and calculated concentrations for all compounds is presented Figure 10a–f displaying a very good correspondence between the model and the experiments. In addition, Figure 10g illustrates that the model is capable of adequately capturing the dependence of hydrogenation rate on the initial substrate concentration.

The degree of explanation was found to be 98.9%. The parameter values shown in Table 2 are well identified apart from the adsorption constant of *trans*-propenylbenzene which is related to its low concentration.

Overall, it can be concluded that the model is capable to capture the kinetic regularities and the concentration profiles for the PdAg_3_ single atom alloy catalyst. As expected, the value *k_5_K*_1*P*1*P*_ is much lower than the corresponding kinetic parameter for hydrogenation of the alkyne to the *cis*-alkene (*k_1_K*_1*P*1*P*_) in line with inferior contribution of the direct hydrogenation to alkane in the overall consumption of the reagent. The ratio between *k_1_K*_1*P*1*P*_ and *k_2_K*_1*P*1*P*_ being ca. 21 reflects high selectivity towards *cis*- propenylbenzene. It is also interesting to note that the adsorption constant for 1-Ph-1-Pr is substantially higher the corresponding value for *cis*-propenylbenzene, which is apparently related to a large difference in the heat of adsorption, as already mentioned above.

## 4. Conclusions

In this study the catalytic performance of Pd_1_Ag_3_/Al_2_O_3_ single-atom alloy catalyst in the liquid phase alkyne hydrogenation was investigated. The catalyst was characterized by TEM, XPS, and TPHD. The single-atom structure of PdAg surface was proved by DRIFTS-CO data demonstrating that Pd_1_ single atom active sites surrounded by Ag atoms are predominant on the catalyst surface, while a contribution of Pd_n_ (*n* ≥ 2) centers is negligible.

It was clearly shown that PdAg SAA catalysts are capable to provide excellent selectivity in hydrogenation of alkynes to the corresponding alkenes not only in the gas-phase, but also in the liquid phase. Thorough studying hydrogenation kinetics of di-substituted non-symmetrical 1-phenyl-1-propyne on Pd_1_Ag_3_/Al_2_O_3_ single-atom alloy catalyst extremely high selectivity to *cis*-propenylbenzene was revealed, being essentially independent on alkyne conversion. Subsequently, direct alkyne hydrogenation to the corresponding alkane over Pd_1_Ag_3_ catalyst can be considered negligible.

Analysis of the literature data and the observed kinetic regularities, namely a negative reaction order in alkyne, the reaction order in hydrogen below unity, as well as independence of selectivity to alkene on alkyne conversion, hydrogen pressure and alkyne concentration, allowed to propose a kinetic model based on these observations and on detailed mechanistic studies on alkyne hydrogenation over SAA catalysts.

In particular, a negative reaction order in alkyne is strong evidence of competitive adsorption of alkyne and hydrogen on Pd_1_ active sites. Subsequently, the reaction mechanism assuming H_2_ and alkyne/alkene adsorption on isolated Pd_1_ sites was taken as the basis for kinetic modeling. The model, developed to describe hydrogenation kinetics in the framework of this mechanism, was based on the Langmuir-Hinshelwood type kinetics with competitive adsorption of the alkyne, the olefin and hydrogen on the same type of isolated Pd_1_ active sites with a fast diffusion of hydrogen between such isolated sites. Numerical data fitting revealed that the kinetic model is able to capture the experimental data showing their excellent correspondence with the calculations. The values of obtained parameters were in line with an insignificant direct transformation of alkyne to alkane and also confirmed much stronger adsorption of the alkyne compared to the main reaction product—*cis*-alkene, which leads to a rapid desorption of the intermediate alkene and, consequently, to an increase in the alkene selectivity.

## Figures and Tables

**Table 1:** Specific surface area of parent Al_2_O_3_ and supported Pd/Al_2_O_3_ and Pd_1_Ag_3_/Al_2_O_3_ catalysts.

Sample	S_BET_, m^2^/g	
	initial	after catalytic test
Al_2_O_3_	59	-
Pd/Al_2_O_3_	57	56
Pd_1_Ag_3_/Al_2_O_3_	55	55

**Table 2:** Values of kinetic parameters for hydrogenation of 1-phenyl-1-propyne over Pd SAA catalyst.

Parameter	Value	Error, %	Units
*k*_1_ *K*_1*P*1*P*_	1.87	26	mol g^−1^ min^−1^
*k*_2_ *K*_1*P*1*P*_	0.089	40.3	mol g^−1^ min^−1^
*k*_3_ *K_C_*	0.05	22.9	mol g^−1^ min^−1^
*k*_4_ *K_T_*	0.17	79	mol g^−1^ min^−1^
*k*_5_ *K*_1*P*1*P*_	0.063	50.7	mol g^−1^ min^−1^
*K*_1*P*1*P*_	76.9	14.7	l/mol

## Data Availability

Data presented in this article is available on request from the corresponding author.

## Supplementary Materials

The following are available online at https://www.mdpi.com/article/10.3390/nano11123286/s1, Figure S1: Product distribution for Pd and Pd1Ag3 catalysts—after the first and after the firth catalytic cycles, Figure S2: Representative TEM micrographs and particle size distribution of Pd and Pd1Ag3 catalysts after the first and after the fifth catalytic cycles.
