# Supplementary material for: Liquid-Phase Hydrogenation of 1-Phenyl-1-propyne on the Pd1Ag3/Al2O3 Single-Atom Alloy Catalyst: Kinetic Modeling and the Reaction Mechanism
Source: Nanomaterials (Basel). 2021 Dec 3;11(12):3286. doi: 10.3390/nano11123286 (PMC8705174; doi:10.3390/nano11123286)
Supplement: Supplementary file 1 [file nanomaterials-11-03286-s001.zip › nanomaterials-1460738-supplementary.pdf]

nanomaterials

Supplementary Material

Liquid-Phase Hydrogenation of 1-phenyl-1-propyne on the Pd_1Ag_3/Al_2O_3 Single-Atom Alloy Catalyst: Kinetic Modeling and the Reaction Mechanism

Alexander V. Rassolov [1], Igor S. Mashkovsky [1,*], Galina N. Baeva [1], Galina O. Bragina [1], Nadezhda S. Smirnova [1], Pavel V. Markov [1], Andrey V. Bukhtiyarov [2], Johan Wärnå [3], Alexander Yu. Stakheev [1,*] and Dmitry Yu. Murzin [3,*]

1 N.D. Zelinsky Institute of Organic Chemistry RAS, Leninsky Prospect 47, 119991 Moscow, Russia; brandon_hitt@mail.ru (A.V.R.); bg305@ioc.ac.ru (G.N.B.); bragina@ioc.ac.ru (G.O.B.); felisine@gmail.com (N.S.S.); markovp@mail.ru (P.V.M.)
2 Synchrotron Radiation Facility SKIF, Boreskov Institute of Catalysis SB RAS, Nikol'skiy Prospekt 1, 630559 Kol'tsovo, Russia; avb@catalysis.ru
3 Laboratory of Industrial Chemistry and Reaction Engineering, Faculty of Science and Engineering, Åbo Akademi University, FI-20500 Turku, Finland; johan.warna@abo.fi
* Correspondence: im@ioc.ac.ru (I.S.M.); st@ioc.ac.ru (A.Y.S.); dmitry.murzin@abo.fi (D.Y.M.)

Citation: Rassolov, A.V.; Mashkovsky, I.S.; Baeva, G.N.; Bragina, G.O.; Smirnova, N.S.; Markov, P.V.; Bukhtiyarov, A.V.; Wärnå, J.; Stakheev, A.Y.; Murzin, D.Y. Liquid-Phase Hydrogenation of 1-phenyl-1-propyne on the Pd_1Ag_3/Al_2O_3 Single-Atom Alloy Catalyst: Kinetic Modeling and the Reaction Mechanism. *Nanomaterials* **2021**, *11*, 3286. https://doi.org/10.3390/nano11123286

Academic Editor: Patricia Concepción

Received: 31 October 2021
Accepted: 29 November 2021
Published: 3 December 2021

Publisher's Note: MDPI stays neutral with regard to jurisdictional claims in published maps and institutional affiliations.

Copyright: © 2021 by the authors. Licensee MDPI, Basel, Switzerland. This article is an open access article distributed under the terms and conditions of the Creative Commons Attribution (CC BY) license (http://creativecommons.org/licenses/by/4.0/).

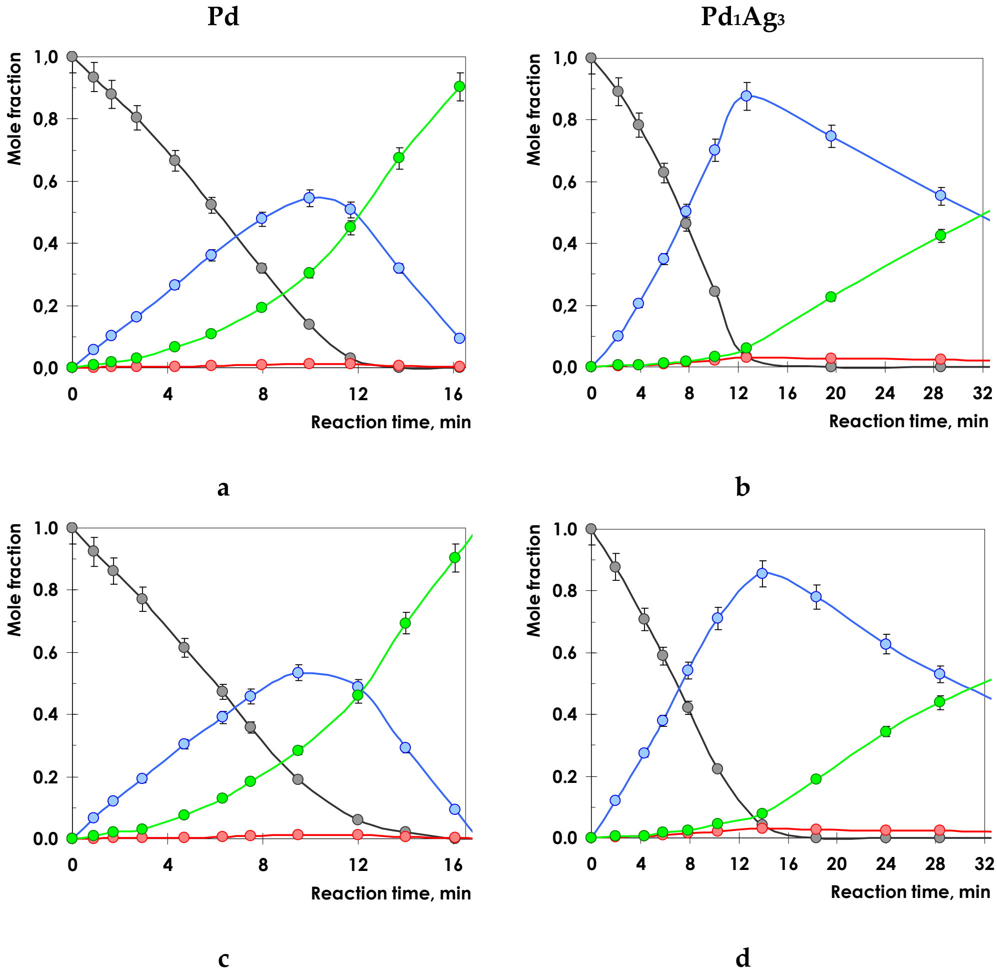

Figure S1. Product distribution for Pd and Pd_1Ag_3 catalysts – after the first (**a**,**b**) and after the firth (**c**,**d**) catalytic cycles. Conditions: T = 25°C, P(H_2) = 5 bar, m(Pd) = 1.5 mg, m(Pd_1Ag_3)= 7.5 mg, V_{C6H14} = 6 mL. The error bars are ±1 standard deviation. Black circles – 1-phenyl-1-propyne, blue circles – *cis*-propenylbenzene, red circles – *trans*–propenylbenzene; green circles – propylbenzene.

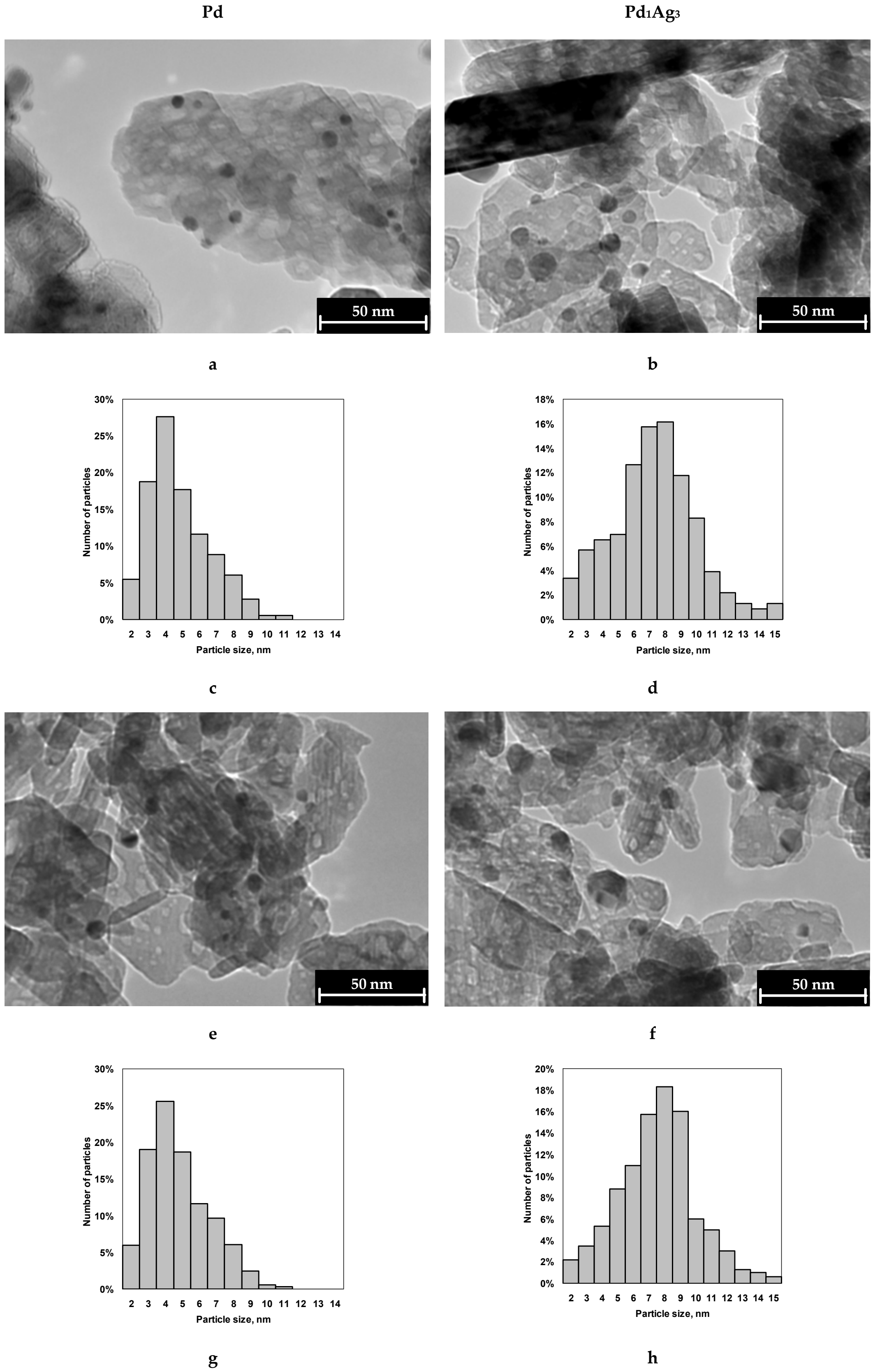

Figure S2. Representative TEM micrographs of Pd and Pd_1Ag_3 catalysts after the first (**a**,**b**) and after the fifth (**e**,**f**) catalytic cycles. Inlets (**c**) and (**d**) illustrates particle size distribution of Pd and Pd_1Ag_3 catalysts after the first catalytic cycle and (**g**) and (**h**) - after the fifth one. The average size of particles is ca. 4 nm for Pd and ca. 8 nm for Pd_1Ag_3.
